# Relationship between Insulin Levels and Nonpsychotic Dementia: A Systematic Review and Meta-Analysis

**DOI:** 10.1155/2017/1230713

**Published:** 2017-12-27

**Authors:** Qiu-xia Pan, Xiao-juan Li, Yue-yun Liu, Fang-fang Wang, Ya-jing Hou, Qing-lai Bian, Wen-qi Qiu, Zhi-yi Yan, You-ming Jiang, Jia-xu Chen

**Affiliations:** School of Basic Medical Science, Beijing University of Chinese Medicine, No. 11 North Third Ring Road, Chaoyang, Beijing 100029, China

## Abstract

**Objectives:**

To explore the relationship between insulin levels and nonpsychotic dementia.

**Methods:**

Six electronic databases (PubMed, Cochrane, SCI, CNKI, VIP, and Wanfang) were searched from January 1, 2007, to March 1, 2017. Experimental or observational studies that enrolled people with nonpsychotic dementia or abnormal insulin levels in which insulin levels or MMSE scores (events in nonpsychotic dementia) were the outcome measures. Random-effects models were chosen for this meta-analysis. Sample size, mean, s.d., and events were primarily used to generate effect sizes (with the PRIMA registration number CRD42017069860).

**Results:**

50 articles met the final inclusion criteria. Insulin levels in cerebrospinal fluid were lower (Hedges' *g* = 1.196, 95% CI = 0.238 to 2.514, and *P* = 0.014), while the levels in peripheral blood were higher in nonpsychotic dementia patients (Hedges' *g* = 0.853 and 95% CI = 0.579 to 1.127), and MMSE scores were significantly lower in the high insulin group than in the healthy control group (Hedges' *g* = 0.334, 95% CI = 0.249 to 0.419, and *P* = 0.000).

**Conclusions:**

Our comprehensive results indicate that blood insulin levels may increase in patients with nonpsychotic dementia.

## 1. Introduction

Dementia is a general term for a decline in mental ability severe enough to interfere with daily life, and it is a common clinical syndrome that is not only a leading cause of death globally but also a burden on families and society. There is no one test to determine if someone has dementia. Doctors diagnose Alzheimer's and other types of dementia based on a careful medical history, a physical examination, laboratory tests, and the characteristic changes in thinking, day-to-day function, and behavior associated with each type. At present, all of the Mini-Mental State Examination (MMSE), Hasegawa's dementia scale, clinical dementia rating, cognitive abilities screening instrument, Alzheimer's disease assessment scale-cognitive section, activity of living, Functional Activities Questionnaire, and clock-drawing test can be used in the diagnosis of dementia, among which, the MMSE is widely used.

As it is harder to determine the exact type of dementia because the symptoms and brain changes of different dementias can overlap, and in some cases, a doctor may diagnose “dementia” and not specify a type. In these cases, we divided the dementia into two aspects (psychotic dementia and nonpsychotic dementia) according to the day-to-day functions, behaviors, and medical history of the patients. Psychotic dementia is caused by mental illness, which means that dementia is caused by depression, schizophrenia, and other mental disorders, while nonpsychotic dementia excludes the dementia caused by mental illness (psychotic dementia) [1], for example, Alzheimer's disease (AD), vascular dementia (VD), and brain lesions. In addition, mild cognitive impairment (MCI) is a “clinical” diagnosis representing a doctor's best professional judgment about the reason for a person's symptoms. MCI causes a slight but noticeable and measurable decline in cognitive abilities, including memory and thinking skills. A person with MCI is at an increased risk of developing Alzheimer's or another dementia. Medical history, assessment of independent function and daily activities, input from a family member or trusted friend, assessment of mental status, in-office neurological examination, evaluation of mood, and laboratory tests were the primary diagnostic route.

Many risk factors are associated with nonpsychotic dementia. These include older age, gender (i.e., female), a low educational level, a low socioeconomic level, certain physical diseases, and a family genetic history. Recently, abnormal insulin levels were shown to be an important factor that influences the occurrence of nonpsychotic dementia. Umegaki et al. [2] performed a prospective study in which cognitive function scores were assessed at baseline and after 3 years in the same patient group. The results suggested that higher insulin and glycol-hemoglobin levels were associated with diabetes-related cognitive dysfunction.

Insulin is an active substance with a variety of biological functions that affect growth and apoptosis in neurons by participating in blood glucose metabolism [3–8]. The discovery of insulin in the brain suggested that insulin not only participates in metabolism and growth but is also involved in higher cognitive functions, such as learning and memory [3–5]. Insulin has been shown to increase the expression of amyloid precursor protein (APP), beta-amyloid 42 (A*β*42), and hyperphosphorylated tau in the hippocampus and frontal cortex in rats [6, 9]. Thus, there may be mechanistic and sequential associations among insulin, impaired cognitive function, and structural AD-like changes.

In humans, aging is associated with decreased metabolic turnover, decreased glucose utilization, and decreased insulin levels because of effects on the regulation of the insulin signaling pathway. Some authors have argued that abnormal insulin metabolism is caused by aging and does not share a causal relationship with nonpsychotic dementia. For example, Burns et al. [8] assessed the relationship between insulin resistance and conditions including cognitive decline and brain atrophy for two years in patients with early AD and nondemented controls. The authors found that insulin was differentially associated with cognitive decline and atrophy in AD and elderly patients. Furthermore, higher levels of peripheral insulin may exert AD-specific benefits, and insulin signaling may be affected by AD-associated systemic physiological changes. Finally, Dorrance et al. [10] supported the notion that insulin resistance exerts positive effects on cerebral vasculature dementia.

Furthermore, exogenous insulin interventions or treatments have been shown to alleviate nonpsychotic dementia symptoms in patients with insulin resistance and improve their Mini-Mental State Examination (MMSE) scores [11, 12]. However, other studies have demonstrated that insulin levels are negatively correlated with nonpsychotic dementia [13]. In addition, some studies have found no relationship between the occurrence of nonpsychotic dementia and insulin levels when patients were compared with healthy control subjects (HCs) [14, 15]. Therefore, both negative and positive correlations have been observed, resulting in controversy regarding whether high insulin levels or low insulin levels cause nonpsychotic dementia. Thus, a meta-analysis of this subject is warranted.

The present meta-analysis was aimed at determining whether nonpsychotic dementia is associated with altered levels of insulin in the blood or cerebrospinal fluid (CSF). We used meta-analytical techniques because they allow data from individual studies to be quantitatively combined to improve the strength of preclinical and clinical evidence.

## 2. Materials and Methods

The meta-analyses performed in this study adhered to the guidelines recommended by the Preferred Reporting Items for Systematic Reviews and Meta-Analysis (PRISMA) statement [16] and are registered with the international prospective register of systematic reviews (PROSPERO). Its PRISMA registration number is CRD42017069860.

### 2.1. Literature Search

Two investigators (Qiu-xia Pan and Xiao-juan Li) independently performed a systematic review of English language publications using the PubMed, SCI, and Cochrane Library databases and Chinese language publications using the CNKI, VIP, and Wanfang databases.

The following search terms were used in the database search: (Dementia or Alzheimer's disease/AD or Mild cognitive impairment/MCI) and (Insulin or insulin signaling pathway). The search was performed to obtain articles published from January 1, 2007, to March 1, 2017. The initial search generated 1287 records, including 474 English and 813 Chinese records. After we screened the titles and abstracts, 263 appropriate articles, including 120 English and 143 Chinese papers, that were related to the present subject were selected for full-text scrutiny. Original studies that reported data on peripheral blood and CSF insulin concentrations in at least two groups of subjects (i.e., dementia or AD and MCI) were included.

### 2.2. Quality Assessment

Studies were appraised for methodological quality using the Newcastle-Ottawa Scale (NOS). The NOS defines the study quality of observational studies (i.e., case-control studies and cross-sectional studies) using an 8-item scale (scored 0–9 points). Two investigators (Qiu-xia Pan and Xiao-juan Li) independently assessed the quality of each paper, and any disagreements were resolved by consensus in a group meeting.

### 2.3. Study Selection

**Figure 1:**
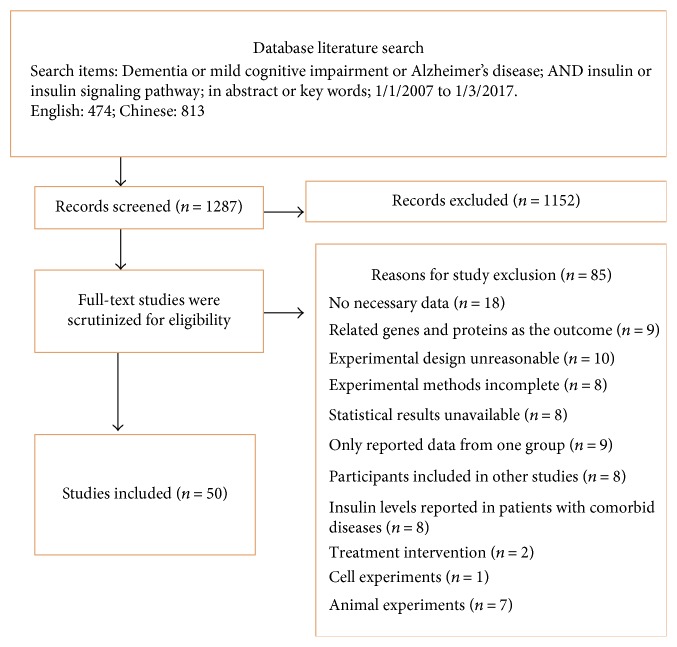
Preferred Reporting Items for Systematic Reviews and Meta-Analysis flowchart of the literature search.

After further evaluating the 135 originally selected articles, 50 high-quality (i.e., had 5–9 points on the NOS, Table 1) [17] articles describing observational studies were included in this study. The remaining 85 articles were excluded for the following reasons: there were no necessary data on insulin concentrations [13, 18–27]; related genes and insulin signaling pathway proteins were the main study outcomes [28–36]; there was no clear description or record of a cognitive function evaluation [37–42]; the design of the experiments was unreasonable for the purpose of this study [43–52]; the experimental methods were incomplete [53–60]; the papers did not provide completely related data [61–66]; data from only one group were reported [2, 67–74]; the papers reported data from the same cohort of patients who were used in another study [75–81]; insulin levels were reported in patients with comorbid diseases (in addition to diabetes, metabolic syndrome and related diseases can also lead to nonpsychotic dementia [82–89]), such as cognitive dysfunction caused by depression or anxiety [85]; serum insulin was measured before and after insulin treatment or other treatments that could influence insulin metabolism in patients with nonpsychotic dementia; only changes in the MMSE scores before and after the treatment were analyzed, while differences between the observation group and the control group were not analyzed [11, 12]; and the studies involved cell experiments [90] or animal experiments [6, 91–96] (for the flowchart, see Figure 1).

### 2.4. Data Extraction

Data regarding sample size, mean, standard deviation (s.d.), events, and *P* values were extracted as the primary outcomes. Two investigators (Qiu-xia Pan and Xiao-juan Li) independently extracted the data, and the results were verified by another team member (Zhi-yi Yan). Any inconsistencies were resolved by consensus. Tables 2 and 3 summarized the included studies and present the demographic and clinical characteristics of the included patients.

### 2.5. Statistical Analysis

Comprehensive Meta-Analysis Version 2 software (Biostat, Englewood, NJ, USA) was used for all statistical analyses. The sample size, mean, s.d., and events were primarily used to generate effect sizes (ESs) (the sample size and *P* value were used in some studies in which the mean, s.d., and events were not available). The ESs were calculated as the standardized mean difference in insulin levels or MMSE scores between groups and converted to Hedges' *g*, which provides an unbiased ES that is adjusted for the sample size. In pooled ESs, 95% confidence intervals (95% CIs) were used to assess significant differences. Random-effects models were chosen for this meta-analysis because we hypothesized that within-study and between-study moderators would result in differences in the true ESs [97]. We excluded one study at a time to determine whether the results were unduly secondary to a particular study. Data regarding the average age and gender distribution of the patients and the classification and severity of cases of nonpsychotic dementia (i.e., MMSE) were also extracted.

Significant differences in heterogeneity across studies were assessed using Cochran's *Q* test [98], and statistical significance was set at *P* < 0.1. Using these parameters, we found that there was between-study heterogeneity. Inconsistencies across studies were identified using the *I* ^2^ index, which evaluates the impact of heterogeneity. *I* ^2^ values of 0.25, 0.50, and 0.75 indicate small, moderate, and high levels of heterogeneity, respectively. We then performed unrestricted maximum-likelihood random-effects metaregressions of the ESs [99] to determine whether covariates, including age, gender distribution (i.e., the proportion of males) and MMSE scores, represented moderators that influenced the ESs. We then performed unrestricted maximum-likelihood random-effects metaregressions of the ESs.

Funnel plots were generated by plotting the ESs against the precision (inverse of the standard error) of each study and used to visually inspect the studies for publication bias. The significance of any observed publication bias was determined using Egger's test [100], which assesses the degree of asymmetry in funnel plots. The classic fail-safe *N* method [101], which is an analysis that results in the number of missing (unpublished) studies that would increase the observed *P* value to >0.05, was also used to investigate publication bias. Statistical significance was set at a *P* value < 0.05 unless otherwise indicated. *P* values < 0.1 were reported as trends.

## 3. Results

### 3.1. Included Studies

In total, 50 high-quality studies (5–9 points) were included in this analysis. These included 206207 participants, and 77 sets of data were summarized for the subsequent analysis. The results were divided into the following three areas according to differences in study objectives: (1) the relationship between insulin levels in the CSF and the risk of nonpsychotic dementia; (2) variability in cognitive function scores (i.e., MMSE scores) and insulin levels in the peripheral blood; and (3) differences in insulin levels between nonpsychotic dementia and nondementia patients (assuming that high insulin levels and nonpsychotic dementia are positively correlated).

### 3.2. Main Associations between CSF Insulin Levels and Nonpsychotic Dementia

**Figure 2:**
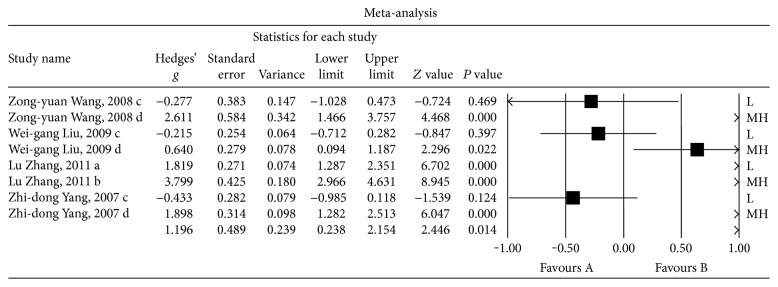
Forest plot of the random-effects meta-analysis of differences in CSF insulin levels between nonpsychotic dementia patients and healthy controls (HC). In all, 8 sets of data encompassing a total of 300 individuals were included. The sizes of the squares are proportional to study weight. CI, confidence interval.

First, we compared the insulin levels (mU/L) in the CSF between patients with nonpsychotic dementia and HCs. Eight sets of data were extracted from 4 studies involving a total of 300 individuals. A random-effects meta-analysis was performed, and the results showed that the nonpsychotic dementia patients had significantly lower CSF insulin levels than were observed in the HCs (Hedges' *g* = 1.196, 95% CI = 0.238 to 2.514, and *P* = 0.014; Figure 2). A sensitivity analysis indicated that our results were significantly influenced by all studies (Additional File 1). In addition, significant heterogeneity was observed among the studies in this meta-analysis (*Q* = 128.753, d.f. = 7, *I* ^2^ = 94.563, and *P* = 0.000).

### 3.3. Main Associations between MMSE Scores and Abnormal Insulin Levels in the Peripheral Blood

**Figure 3:**
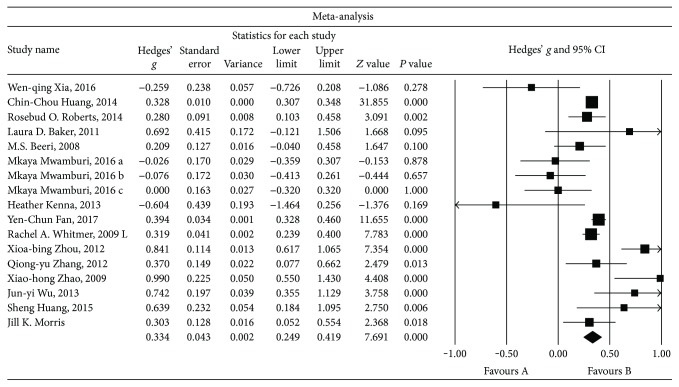
Forest plot of the random-effects meta-analysis of differences in MMSE scores between abnormal insulin levels and HC subjects. In all, 17 sets of data encompassing a total of 197114 individuals were included. The sizes of the squares are proportional to study weight. CI, confidence interval.

Second, we compared the MMSE scores and the incidence of nonpsychotic dementia between patients with abnormal levels of insulin and HCs. Sixteen sets of data were extracted from 15 studies involving a total of 197114 individuals. A random-effects meta-analysis was performed, and the results showed that high insulin levels were associated with a higher risk of nonpsychotic dementia than was observed in the HCs (Hedges' *g* = 0.334, 95% CI = 0.249 to 0.419, and *P* = 0.000; Figure 3). A sensitivity analysis indicated that our results were not unduly influenced by any particular study (Additional File 2). Furthermore, high heterogeneity was observed among the studies in this meta-analysis (*Q* = 65.130, d.f. = 16, *I* ^2^ = 75.434, and *P* = 0.000).

### 3.4. Main Associations between Insulin Levels and Nonpsychotic Dementia

**Figure 4:**
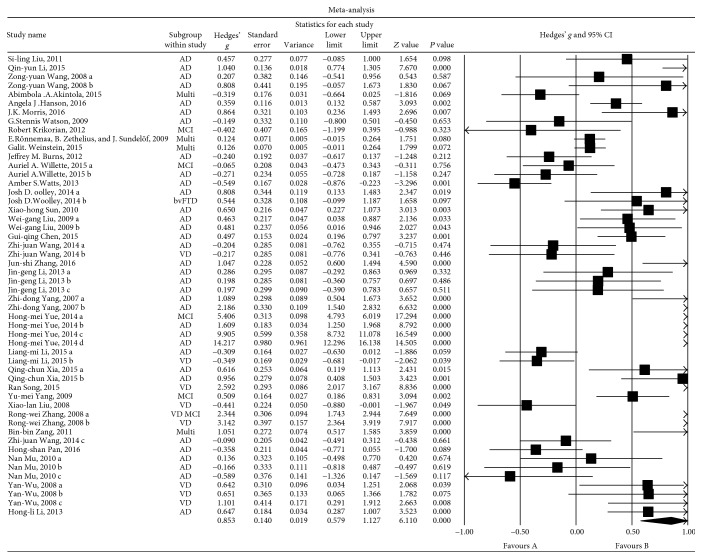
Forest plot of the random-effects meta-analysis of differences in blood insulin concentrations between nonpsychotic dementia patients and HCs. In all, 52 sets of data encompassing a total of 8931 individuals were included. The sizes of the squares are proportional to study weight. CI, confidence interval.

Third, we compared the levels of insulin (mU/L) in the peripheral blood between patients with nonpsychotic dementia and HCs. Fifty-two sets of data were extracted from 36 studies involving a total of 8931 individuals. A random-effects meta-analysis was performed, and the results showed that the nonpsychotic dementia patients had significantly higher insulin levels than were observed in the HCs (Hedges' *g* = 0.853, 95% CI = 0.579 to 1.127, and *P* = 0.000; Figure 4). A sensitivity analysis indicated that our results were not unduly influenced by any particular study (Additional File 3). However, significant heterogeneity was observed among the studies in this meta-analysis (*Q* = 1184.942, d.f. = 51, *I* ^2^ = 95.696, and *P* = 0.000).

### 3.5. Investigation of Heterogeneity

Fourth, to identify the potential sources of the heterogeneity observed in this meta-analysis, we performed subgroup analyses primarily by considering the sources of the samples (i.e., serum or plasma) and the reported medications used in the patients. We then performed subgroup analyses according to the class of the samples (i.e., VD or AD) and the degree of nonpsychotic dementia in the patients.

The subgroup analysis showed that compared with the levels observed in the HCs, serum insulin levels were significantly higher in the patients with nonpsychotic dementia (25 sets of data were extracted from 17 studies; Hedges' *g* = 1.482, 95% CI = 0.909 to 2.056, and *P* = 0.000), and the same trend was observed in the plasma (13 sets of data were extracted from 9 studies; Hedges' *g* = 0.445, 95% CI = 0.154 to 0.736, and *P* = 0.003). Compared with the plasma group, the serum group had significantly greater ESs in the nonpsychotic dementia patients with higher insulin levels. High levels of heterogeneity were observed among studies that included serum (*Q* = 1011.719, d.f. = 25, *I* ^2^ = 97.529, and *P* = 0.000) or plasma (*Q* = 79.543, d.f. = 12, *I* ^2^ = 84.914, and *P* = 0.000) insulin levels. Subgroup analyses of studies involved patients who did not take any drugs, who took drugs without effect on glucose and lipid metabolism, and who are on drug withdrawal that lasted longer than 2 weeks. The subjects were then grouped, and 10 sets of data were extracted from 8 studies involving a total of 731 individuals. A random-effects meta-analysis showed that insulin levels were not significantly different between the nonpsychotic dementia patients and HCs (Hedges' *g* = 0.194, 95% CI = −0.251 to 0.640, and *P* = 0.393). High levels of heterogeneity were also found (*Q* = 76.093, d.f. = 9, *I* ^2^ = 88.172, and *P* = 0.000).

### 3.6. Subgroup Analyses

#### 3.6.1. Associations between Insulin Levels and the VD Group

**Figure 5:**
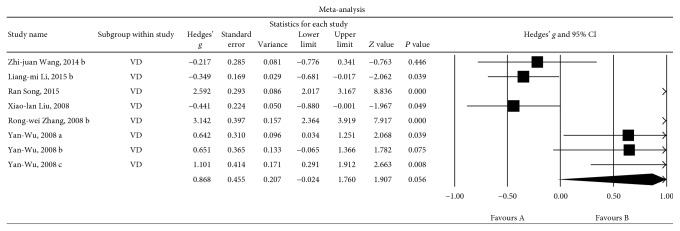
Forest plot of the random-effects meta-analysis of differences in blood insulin concentrations between VD patients and HC subjects. In all, 8 sets of data encompassing a total of 488 individuals were included. The sizes of the squares are proportional to study weight. CI, confidence interval.

Eight sets of data were extracted from 6 studies involving a total of 488 individuals. A random-effects meta-analysis was performed, and the results showed that the risk of VD was higher in patients with high insulin levels than in the HCs (Hedges' *g* = 0.868, 95% CI = −0.024 to 1.760, and *P* = 0.056; Figure 5). A sensitivity analysis indicated that two studies could have influenced this outcome (Additional File 4). This was not surprising because the *P* values of the meta-analysis outcomes were only slightly higher than 0.05. Significant heterogeneity was observed among the studies in this meta-analysis (*Q* = 145.849, d.f. = 8, *I* ^2^ = 95.201, and *P* = 0.000).

#### 3.6.2. Associations between Insulin Levels and the AD Group

**Figure 6:**
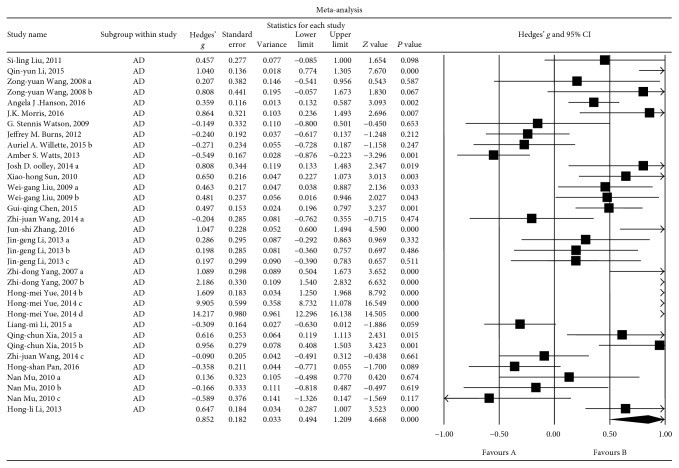
Forest plot of the random-effects meta-analysis of differences in blood insulin concentrations between AD patients and HC subjects. In all, 34 sets of data encompassing a total of 8407 individuals were included. The sizes of the squares are proportional to study weight. CI, confidence interval.

Thirty-four sets of data were extracted from 24 studies involving a total of 8407 individuals. A random-effects meta-analysis was performed, and the results showed that the nonpsychotic dementia patients had significantly higher insulin levels than were observed in the HCs (Hedges' *g* = 0.852, 95% CI = 0.494 to 1.209, and *P* = 0.000; Figure 6). A sensitivity analysis indicated that these results were not unduly influenced by any particular study (Additional File 5). However, high levels of heterogeneity among the studies were observed in this meta-analysis (*Q* = 678.926, d.f. = 33, *I* ^2^ = 95.139, and *P* = 0.000).

Fewer than 5 studies focused on the relationship between abnormal insulin levels and nonpsychotic dementia types other than AD and VD. Hence, we compared only the relationships between abnormal insulin levels and AD or VD because these two subgroups showed high heterogeneity. Thus, we next sought to determine whether there are differences in blood insulin levels between HCs and patients with MCI, mild (or light) nonpsychotic dementia (L, MMSE scores ≥ 16) and moderate or heavy nonpsychotic dementia (MH, MMSE scores < 16).

#### 3.6.3. Associations between Insulin Levels and MCI

**Figure 7:**
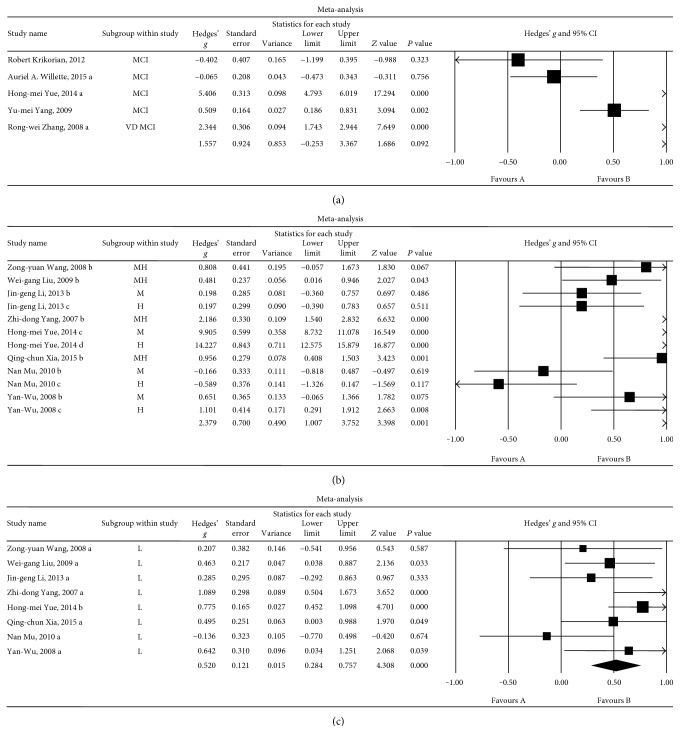
Forest plot of the random-effects meta-analysis of differences in blood insulin concentrations between patients with different severities of dementia and HC subjects. These 3 pictures in (a), (b), and (c) represent a comparison of insulin levels between HC subjects and MCI, mild (L), and moderate to severe (MH) dementia patients, respectively.

Five sets of data were extracted from 5 studies involving a total of 4653 individuals. A random-effects meta-analysis was performed, and the results showed that there were no significant differences in blood insulin levels between the MCI and HC groups (Hedges' *g* = 1.557, 95% CI = −0.253 to 3.367, and *P* = 0.092; Figure 7). A sensitivity analysis was not performed because the number of studies was too small. However, we identified significant heterogeneity among the studies (*Q* = 264.128, *I* ^2^ = 98.486, and *P* < 0.001).

#### 3.6.4. Associations between Insulin Levels and Patients with Mild (or Light) Nonpsychotic Dementia

Eight sets of data were extracted from 8 studies involving a total of 543 individuals. A random-effects meta-analysis was performed, and the results showed that mild nonpsychotic dementia patients had significantly higher insulin levels than were observed in the HCs (Hedges' *g* = 0.520, 95% CI = 0.284 to 0.757, and *P* = 0.000; Figure 7). A sensitivity analysis indicated that these results were not unduly influenced by any particular study (Additional File 6). Furthermore, low heterogeneity was observed among the studies in this meta-analysis (*Q* = 11.531, d.f. = 7, *I* ^2^ = 39.293, and *P* = 0.117), demonstrating that the differences among the groups made a negligible contribution to this heterogeneity.

#### 3.6.5. Associations between Insulin Levels and Patients with Moderate or Heavy Nonpsychotic Dementia

Twelve sets of data were extracted from 8 studies involving a total of 610 individuals. A random-effects meta-analysis was performed, and the results showed that the middle and heavy nonpsychotic dementia patients had significantly higher insulin levels than were observed in the HCs (Hedges' *g* = 2.379, 95% CI = 1.007 to 3.752, and *P* = 0.001; Figure 7). A sensitivity analysis indicated that the results were not unduly influenced by any particular study (Additional File 7). However, significant heterogeneity was observed among the studies in this meta-analysis (*Q* = 531.782, d.f. = 11, *I* ^2^ = 97.931, and *P* = 0.000).

The results of the three subgroup analyses demonstrated that the high heterogeneity observed for increased insulin levels in the peripheral blood in patients with nonpsychotic dementia may have been caused by the severity of the dementia as follows: the greater the severity of nonpsychotic dementia, the higher the ESs of peripheral blood insulin levels. Compared with those of the HC group, the insulin levels in the MCI group were not correlated with the MCI.

In a series of metaregression analyses, we assessed whether gender or continuous variables, such as age, could explain the observed between-study differences. Gender was found to have a moderating effect (*P* = 0.29), but age did not significantly contribute to heterogeneity (*P* < 0.05).

Overall, our results indicated that the significant heterogeneity observed in the patients with increased insulin and nonpsychotic dementia could have been caused by a variety of factors, such as gender, sampling differences, and the severity of nonpsychotic dementia.

### 3.7. Publication Bias

In the abnormal insulin group, no significant publication bias was detected among the studies in a visual inspection of funnel plots (Additional File 8). These results were confirmed in Egger's tests (*t* = 0.007, d.f. = 15, and *P* = 0.497). Moreover, the nonpsychotic dementia group and AD group showed slight publication biases and had the following Egger's test values: for insulin, *t* = 3.26, d.f. = 50, and *P* = 0.001 (Additional File 9); and for AD, *t* = 2.399, d.f. = 32, and *P* = 0.011 (Additional File 10). Because the ESs of small sample studies estimate a large amount of variation, the appearance of extreme ES values is more likely to emerge in small sample studies than in large sample studies. This publication bias was not statistically significant, and we therefore applied Duval and Tweedie's trim and fill method in further analyses.

The classic fail-safe *N* was used to assess publication bias, and the results revealed that 4805 missing studies would be required for insulin and 1809 missing studies would be required for AD to achieve *P* values of >0.05. These results support the notion that the observed publication bias was unlikely to have caused the positive results of our meta-analysis.

## 4. Discussion

Many studies have focused on the changes that occur in the Pi3k-Akt insulin signaling pathway in nonpsychotic dementia patients. The Pi3k-Akt insulin signaling pathway is an important pathway in insulin metabolism and is also involved in growth and development, metabolism, and vital cognitive activities. Therefore, it is important to determine whether insulin plays a specific role in nonpsychotic dementia. Whether circulating levels of insulin are altered in nonpsychotic dementia patients has been controversial for a long time. Hence, the significance of insulin levels to the etiology of nonpsychotic dementia etiology was not fully known. The results of this study provide strong clinical evidence supporting the notion that nonpsychotic dementia is associated with increased levels of circulating insulin and decreased levels of insulin in the CSF. These results provide new insights into a potential molecular pathway that confers vulnerability to the development of nonpsychotic dementia. A strength of this study is that it is based on a large amount of data and a sufficient number of studies related to nonpsychotic dementia.

We explored the relationship between insulin levels and the occurrence of nonpsychotic dementia from three different perspectives. Our results show that CSF insulin levels are lower in patients with nonpsychotic dementia than in HCs, while insulin levels in the peripheral blood may be higher. Additionally, high insulin levels in the peripheral blood were associated with lower scores in cognitive functions. Because the number of studies that included a CSF group, a VD group, an MCI patient group, an L group, and an MH group was small, our results only reveal that there is a correlation between insulin levels and nonpsychotic dementia in these groups. The results suggest that insulin levels are higher in the peripheral blood in VD, AD, L, and MH patients while CSF insulin levels are lower in nonpsychotic dementia patients. The ES was higher in the VD group than in the AD group and higher in the MH group than in the L group.

Our results show that there was no difference in insulin levels between MCI patients and HCs. Eight sets of data obtained from 4 studies support the finding that there is a relationship between CSF insulin levels and the severity of nonpsychotic dementia. Because the number of enrolled patients was small, the present results show that CSF insulin levels are lower in patients with severe nonpsychotic dementia, and there were no significant differences between MCI patients and HCs. Moreover, when the effects of drugs on insulin metabolism were excluded, a subgroup analysis showed that there was no correlation between nonpsychotic dementia and insulin levels. Therefore, the following two possibilities are considered. First, because MMSE scores were significantly lower in the high insulin subjects than in the HC group, the fact that there was no correlation may be related to the small sample size. Hence, as the study size increased, the trend toward an increase in serum insulin levels in the MCI patients might reach significance, and insulin levels are more likely to vary with the severity of the nonpsychotic dementia. Second, because the severity of nonpsychotic dementia increased with the ages of the affected patients, insulin levels may simply increase with age and may not be a key factor in the pathology of nonpsychotic dementia.

Several studies [44–46] have described insulin levels and MMSE scores in nonpsychotic dementia patients without diabetes, type 2 diabetes patients without dementia, and patients with nonpsychotic dementia and type 2 diabetes, and these studies have reported that there are no significant differences in the ages or genders of these patients. Insulin levels increased in the nonpsychotic dementia patients, and cognitive function scores decreased in subjects with high insulin levels. Further investigations of insulin levels in MCI patients are required to confirm these results.

In this meta-analysis, we found various levels of between-study heterogeneity in the outcomes. Although we attempted to adjust for potential confounders, none of the theoretically relevant categorical or continuous variables that were tested explained the observed heterogeneity. A subgroup analysis showed that serum insulin levels (as opposed to plasma levels) were significantly higher in nonpsychotic dementia patients than in HC subjects and that VD insulin levels (as opposed to AD levels) were significantly higher in nonpsychotic dementia patients than in HC subjects. However, the high level of between-study heterogeneity was not lower in these subgroup analyses. MH insulin levels (but not L levels) were significantly higher in nonpsychotic dementia patients than in HC subjects, and low heterogeneity was found in the L group analysis. The high level of between-study heterogeneity was not reduced in the MH and drug intervention elimination groups. This high level of between-study heterogeneity may have been caused by the differences in the samples (i.e., according to gender, age, or sample type), the type of nonpsychotic dementia, the degree of nonpsychotic dementia, or the drug intervention. In addition, hunger, cold, exogenous diet stimulation, endocrine diseases, and the selection of inspection methods and reagents could potentially have caused an abnormal secretion of insulin. Hence, potential confounders that could contribute to the observed between-study heterogeneity were present in a variety of the analyzed studies and may have been related to patient conditions during sample extraction. Because insulin levels that are determined using blood samples can be affected by handling time and the state of the research subjects, studies that include patients who provide samples while on an empty stomach and who are not affected by other endocrine factors may be required to further investigate whether these factors contributed to the variance observed among the studies in this analysis.

## 5. Conclusions

Overall, CSF insulin levels were lower in nonpsychotic dementia patients (enrolled sample number < 10, more experimental data are needed to support this finding). The high insulin subjects had significantly lower MMSEs and nonpsychotic dementia events than were observed in the HCs, and blood insulin levels were significantly higher in nonpsychotic dementia patients than in HCs. These results indicate that insulin levels are an important indicator that may be useful in clinical diagnoses of nonpsychotic dementia.

## Figures and Tables

**Table 1:** Quality assessment of the included studies according to the Newcastle-Ottawa Scale (NOS)^∗^.

Study names	S1	S2	S3	S4	C1a	C1b	E1a	E1b	E2	E3	Total points
Si-ling Liu, 2011	1	1	1	1	0	1	1	0	0	0	6
Qin-yun Li, 2015	1	1	1	1	0	1	1	0	1	0	7
Zong-yuan Wang, 2008	1	1	1	1	0	1	1	0	1	0	7
Abimbola A. Akintola, 2015	0	1	1	0	0	1	1	0	1	0	5
Angela J. Hanson, 2016	1	1	0	0	1	0	1	0	1	0	5
J.K. Morris, 2016	1	1	0	0	0	1	1	0	1	0	5
G. Stennis Watson, 2009	1	1	1	1	0	1	1	0	1	0	7
Robert Krikorian, 2012	1	1	0	0	0	1	1	0	1	0	5
E. Rönnemaa, B. Zethelius, and J. Sundelöf, 2009	1	1	0	1	1	0	0	1	1	0	6
Galit Weinstein, 2015	0	1	1	1	0	1	1	0	1	0	6
Jeffrey M. Burns, 2012	0	1	1	1	0	1	1	0	1	0	6
Auriel A. Willette, 2015	0	1	1	1	0	1	1	0	1	0	6
Amber S. Watts, 2013	1	1	1	1	1	1	1	0	1	1	9
Josh D. Woolley, 2014	1	1	0	0	1	0	1	0	1	1	6
Xiao-hong Sun, 2010	1	1	0	1	1	0	1	0	1	0	6
Lu Zhang, 2011	1	1	0	1	1	0	1	0	1	0	6
Wei-gang Liu, 2009	1	1	0	0	1	0	1	0	1	0	5
Gui-qing Chen, 2015	1	1	0	1	1	0	1	0	1	1	7
Zhi-juan Wang, 2014a	1	1	0	1	1	0	1	0	1	0	6
Jun-shi Zhang, 2016	1	0	0	1	1	1	1	0	1	1	7
Jin-geng Li, 2013	1	1	0	1	1	1	1	0	1	1	8
Zhi-dong Yang, 2007	1	0	1	1	0	1	1	0	1	0	6
Hong-mei Yue, 2014	1	1	0	1	1	1	1	0	1	0	7
Liang-mi Li, 2015	1	1	1	1	1	1	1	0	1	0	8
Qing-chun Xia, 2015	1	1	0	1	1	1	1	1	1	0	8
Ran Song, 2015	1	1	0	1	1	1	1	0	1	1	8
Yu-mei Yang, 2009	1	0	0	1	1	1	1	0	1	0	6
Xiao-lan Liu, 2008	1	1	0	1	1	1	1	0	1	1	8
Rong-wei Zhang, 2008	1	1	0	0	1	0	1	0	1	0	5
Bin-bin Zang, 2011	1	1	0	1	1	1	1	0	1	0	7
Zhi-juan Wang, 2014b	1	1	1	1	1	0	1	0	1	0	7
Hong-shan Pan, 2016	1	1	0	1	1	0	1	0	1	0	6
Nan Mu, 2010	1	1	1	1	1	0	1	0	1	0	7
Yan-Wu, 2008	1	1	0	1	1	0	1	0	1	0	6
Hong-li Li, 2013	1	1	0	1	1	0	1	0	1	0	6
Jill K. Morris, 2014	1	0	0	0	1	1	1	0	1	1	6
Wen-qing Xia, 2016	1	1	1	1	1	0	1	0	1	0	7
Chin-Chou Huang, 2014	1	1	1	1	0	1	1	0	1	0	7
Rosebud O. Roberts, 2014	1	1	1	0	0	1	1	0	1	0	6
Mkaya Mwamburi, 2016^∗^	1	1	0	1	0	0	1	1	1	0	6
Heather Kenna, 2013	1	1	1	1	1	0	1	0	1	0	7
Yen-Chun Fan, 2017	1	1	1	1	0	1	1	0	1	0	7
Laura D. Baker, 2011	0	1	1	1	0	1	1	0	1	0	6
M.S. Beeri, 2008	0	1	1	1	0	1	1	0	1	0	6
Hannah Bruehl, 2009	1	1	1	1	1	1	1	1	1	0	9
Xiao-bing Zhou, 2012	0	1	0	1	1	0	1	0	1	0	5
Qiong-yu Zhang, 2012	1	1	0	0	1	1	1	0	1	0	6
Xiao-hong, Zhao, 2009	1	1	0	1	1	1	1	0	1	0	7
Jun-yi Wu, 2013	1	1	0	1	1	1	1	0	1	0	7
Sheng Huang, 2015	1	1	0	1	1	1	1	0	1	0	7

*Note*. S1: eligibility criteria; S2: representativeness of the cases; S3: community controls; S4: the controls had no history of disease (endpoint); C1a: important factor basis between two groups; C1b: study controls for additional factor basis between two groups; E1a: secure record of exposure; E1b: structured interview where blind to case about the exposure; E2: same method of ascertainment for cases and controls; E3: nonresponse rate. ^∗^Cross-sectional study.

**Table 2:** Baseline subject characteristics (nonpsychotic dementia and HCs).

Names (IN) mmol/l	Age		Edu	Sex		Number		Nonpsychotic dementia		HC		Sample	State	Drug	*P* value
	Ex	HC		Men	Women	Ex	HC	Mean	s.d.	Mean	s.d.				
Si-ling Liu, 2011 AD	72	72	11.2	34	19	23	30	—	—	—	—	Serum	Fast	—	*P* < 0.05 H
Qin-yun Li, 2015 AD	77	75	10.1	126	120	126	120	11.78	6.6	6.28	3.36	Serum	Fast	No	*P* = 0.04 H
Zong-yuan Wang, 2008 a AD L	70	72	—	—	—	14	12	5.7	6.9	4.5	3.5	Plasma	Fast	No	*P* < 0.05 H
Zong-yuan Wang, 2008 b AD (MH)	70	72	—	—	—	9	12	10.3	9.8	4.5	3.5	Plasma	Fast	No	*P* < 0.05 H
Zong-yuan Wang, 2008 c AD L	70	72	—	—	—	14	12	3.4	0.3	3.3	0.4	CSF	Fast	No	*P* > 0.05
Zong-yuan Wang, 2008 d AD (MH)	70	72	—	—	—	9	12	2.4	0.2	3.3	0.4	CSF	Fast	No	*P* < 0.05 L
Abimbola A. Akintola, 2015 multi	66	66	—	62	70	75	57	—	—	—	—	Plasma	Fast	No	*P* = 0.07
Angela J. Hanson, 2016 AD	68	72	—	163	156	199	120	15.6	—	11.7	—	Plasma	Fast	—	*P* = 0.0005 H
J.K. Morris, 2016 AD L	71	72	17	22	29	14	37	8.73	7.8	4.19	3.8	Plasma	Fast	Yes	*P* = 0.023 H
G. Stennis Watson, 2009 AD	76	74	15	13	22	16	19	8.2	4.3	8.9	4.8	Plasma	Fast	No	*P* > 0.05
Robert Krikorian, 2012 MCI	71	68	15	—	—	11	12	14.4	6	16.9	6	—	Fast	No	*P* = 0.40
E. Rönnemaa, B. Zethelius, and J. Sundelöf, 2009 multi	71	71	—	—	—	257	868	—	1.0	—	0.96	Plasma	Fast	Yes	*P* = 0.04 H
Galit Weinstein, 2015 multi	47	47	—	2156	1782	215	3723	—	—	—	—	—	Fast	Yes	*P* = 0.036 H
Jeffrey M. Burns, 2012 AD	75	73	16	59	50	48	61	8.4	5.4	7.2	4.6	Serum	Fast	—	*P* = 0.19
Auriel A. Willette, 2015 a MCI	76	76	15	95	185	194	26	2.78	3.08	2.98	3.10	Plasma	Fast	—	*P* = 0.589
Auriel A. Willette, 2015 AD b	76	76	15	95	185	60	26	2.41	1.46	2.98	3.10	Plasma	Fast	—	*P* = 0.589
Amber S. Watts, 2013 AD	75	74	—	61	87	75	73	—	—	—	—	Serum	Fast	—	*P* = 0.001^∗^
Josh D. Woolley, 2014 a AD	59	57	15	16	19	17	18	—	—	—	—	Serum	-3 min	—	*P* < 0.01 H
Josh D. Woolley, 2014 b (bvFTD)	59	57	15	18	19	19	18	—	—	—	—	Serum	−30 min	—	*P* < 0.05 H
Xiao-hong Sun, 2010 AD	—	—	—	32	58	45	44	12.36	8.627	6.541	9.120	Serum	Fast	—	*P* < 0.05 H
Lu Zhang, 2011 a AD L	72	72	—	59	44	41	35	1.07	0.21	1.51	0.27	CSF	—	—	*P* < 0.05 L
Lu Zhang, 2011 b AD MH	72	72	—	59	44	27	35	0.68	0.11	1.51	0.27	CSF	—	—	*P* < 0.01 L
Wei-gang Liu, 2009 a AD L	71	71	—	66	59	31	70	7.9	2.0	6.8	2.5	—	Fast	—	*P* < 0.01 H
Wei-gang Liu, 2009 b AD MH	71	71	—	66	59	24	70	8.0	2.4	6.8	2.5	—	Fast	—	*P* < 0.01 H
Wei-gang Liu, 2009 c AD L	71	71	—	45	38	27	35	0.27	0.08	0.29	0.10	CSF	—	—	*P* > 0.05
Wei-gang Liu, 2009 d AD MH	71	71	—	45	38	21	35	0.22	0.12	0.29	0.10	CSF	—	—	*P* < 0.05 L
Gui-qing Chen, 2015 AD	78	78	7	94	81	95	80	9.758	7.42	6.384	5.89	Serum	Fast	—	*P* = 0.023 H
Zhi-juan Wang, 2014 a AD	67	67	—	28	22	30	20	7.94	6.81	9.25	5.51	Serum	Fast	—	*P* > 0.05
Zhi-juan Wang, 2014 b VD	67	67	—	29	23	30	20	7.89	6.55	9.25	5.51	Serum	Fast	—	*P* > 0.05
Jun-shi Zhang, 2016 AD	76	76	—	47	39	43	43	11.79	6.62	6.25	3.34	Serum	Fast	—	*P* < 0.05 H
Jin-geng Li, 2013 a AD L	73	73	7	39	46	18	30	10.667	10.51	6.793	14.75	—	Fast	—	*P* < 0.01 H
Jin-geng Li, 2013 b AD M	73	73	7	39	46	20	30	9.203	5.36	6.793	14.75	—	Fast	—	*P* < 0.01 H
Jin-geng Li, 2013 c AD H	73	73	7	39	46	17	30	9.324	7.48	6.793	14.75	—	Fast	—	*P* < 0.01 H
Jin-geng Li, 2013 d AD (total)	73	73	7	39	46	55	30	9.718	10.31	6.793	14.75	—	Fast	—	*P* < 0.01 H
Zhi-dong Yang, 2007a AD L	—	—	—	36	43	21	31	16.1	3.7	11.6	4.3	Plasma	Fast	—	*P* < 0.05 H
Zhi-dong Yang, 2007 b AD MH	—	—	—	36	43	27	31	22.1	5.2	11.6	4.3	Plasma	Fast	—	*P* < 0.05 H
Zhi-dong Yang, 2007 c AD L	—	—	—	36	43	21	31	0.98	0.33	1.12	0.31	CSF	Fast	—	*P* > 0.05
Zhi-dong Yang, 2007 d AD MH	—	—	—	36	43	27	31	0.63	0.17	1.12	0.31	CSF	Fast	—	*P* < 0.05 L
Hong-mei Yue, 2014 a MCI	72	72	10	—	—	116	76	19.90	1.62	11.15	1.60	Serum	Fast	—	*P* < 0.05 H
Hong-mei Yue, 2014 b AD L	72	72	10	—	—	81	76	17.90	5.6	11.15	1.6	Serum	Fast	—	*P* < 0.05 H
Hong-mei Yue, 2014 c AD M	72	72	10	—	—	72	76	27.08	1.6	11.15	1.6	Serum	Fast	—	*P* < 0.05 H
Hong-mei Yue, 2014 d AD H	72	72	10	—	—	34	76	34.10	1.61	11.15	1.6	Serum	Fast	—	*P* < 0.05 H
Liang-mi Li, 2015 a AD	73	73	—	117	103	80	70	7.94	0.83	8.23	1.04	Serum	Fast	—	*P* > 0.05
Liang-mi Li, 2015 b VD	73	73	—	117	103	70	70	7.89	0.89	8.23	1.04	Serum	Fast	—	*P* > 0.05
Qing-chun Xia, 2015 a AD L	70	70	—	27	63	34	30	30.29	20.00	20.15	20.50	Plasma	Fast	No	*P* < 0.05 H
Qing-chun Xia, 2015 b AD MH	70	70	—	27	63	26	30	40.29	21.10	20.15	20.50	Plasma	Fast	No	*P* < 0.05 H
Ran Song, 2015 VD	65	65	—	53	32	39	46	8.88	0.31	4.97	2.01	Serum	Fast	Yes	*P* < 0.001 H
Yu-mei Yang, 2009 MCI	63	63	—	81	114	51	144	10.50	—	8.10	—	—	Fast	—	*P* = 0.001 H
Xiao-lan Liu, 2008 VD	74	74	—	88	32	40	40	10.01	—	8.7	—	—	Fast	No	*P* > 0.05
Rong-wei Zhang, 2008 a MVCI	73	73	—	56	38	37	34	13.5	1.4	10.40	1.2	Serum	Fast	—	*P* < 0.05
Rong-wei Zhang, 2008 b VD	73	73	—	56	38	23	34	15.4	2.0	10.40	1.2	Serum	Fast	—	*P* < 0.05 H
Bin-bin Zang, 2011 multi	76	76	—	33	27	30	30	6.42	0.58	5.53	1.03	Serum	Fast	—	*P* < 0.05 H
Zhi-juan Wang, 2014 c AD	74	74	—	69	35	68	36	7.82	6.46	8.37	5.31	Serum	Fast	—	*P* > 0.05
Hong-shan Pan, 2016 AD	69	69	—	50	40	45	45	7.7	4.5	9.4	4.9	—	Fast	No	*P* > 0.05
Nan Mu 2010, a AD L	81	81	—	—	—	13	32	25.38	53.11	20.75	21.51	Serum	Fast	—	*P* = 0.605
Nan Mu, 2010 b AD M	81	81	—	—	—	12	32	17.38	14.89	20.75	21.51	Serum	Fast	—	*P* = 0.605
Nan Mu, 2010 c AD H	81	81	—	—	—	9	32	9.11	6.03	20.75	21.51	Serum	Fast	—	*P* = 0.605
Yan-Wu, 2008 a VD L	62	62	—	—	—	35	15	12.89	4.52	10.01	4.15	—	Fast	—	*P* < 0.05 H
Yan-Wu, 2008 b VD M	62	62	—	—	—	15	15	13.13	5.13	10.01	4.15	—	Fast	—	*P* < 0.01 H
Yan-Wu, 2008 c VD H	62	62	—	—	—	11	15	15.32	5.31	10.01	4.15	—	Fast	—	*P* < 0.01 H
Hong-li Li, 2013 AD	74	74	—	56	69	55	70	5.75	4.23	3.71	1.87	Serum	Fast	—	*P* < 0.05 H

*Note*. Ex: experimental group; HC: healthy control group; HCs: healthy control subjects; Edu: average years of education of all participants; state: sampling time; drug: participants received or did not receive medication interventions during the course of the study; AD: Alzheimer's disease; VD: vascular dementia; multi: nonpsychotic dementia caused by multiple diseases; MCI: mild cognitive impairment; L/M/H: different severities (light/mild, middle, and heavy) of nonpsychotic dementia, respectively; MVCI: mild cognitive impairment caused by vascular disease; H: high insulin levels in patients in the experimental group; L: low insulin levels in patients in the experimental group. ^∗^This study included a comparison of log values, and although the *P* value was <0.05, the actual analysis showed that insulin levels were not associated with events in nonpsychotic dementia.

**Table 3:** Baseline characteristics of the subjects (abnormal insulin levels and HCs).

Names (events/MMSEs)	Age		Edu	Sex		Number		Ex		HC		*P* value
	Ex	HC		Men	Women	Ex	HC	Mean	s.d.	Mean	s.d.	
								Events		Events		
Wen-qing Xia, 2016	59.5	56.2	10.3	32	38	38	32	28.8	1.1	29.1	1.2	*P* < 0.001 H
Chin-Chou Huang, 2014	79	79	—	73941	68803	71433	71311	8572		4992		*P* < 0.001 H
Rosebud O. Roberts, 2014	80	79	14	423	326	154	595	—	—	—	—	*P* = 0.914
Mkaya Mwamburi, 2016 a	75	80	12	—	—	70	67	25.3	3.4	25.4	4.2	*P* = 0.914
Mkaya Mwamburi, 2016 b	75	80	12	—	—	67	67	25.1	3.6	25.4	4.2	*P* = 0.914
Mkaya Mwamburi, 2016 c	75	80	12	—	—	83	67	25.4	3.2	25.4	4.2	*P* > 0.05
Heather Kenna, 2013	59	57	16	—	—	10	10	28.9	0.9	29.5	1.0	*P* < 0.001 H
Yen-Chun Fan, 2017	53	53	—	27360	24220	10316	41246	413		825		*P* < 0.05 H
Laura D. Baker, 2011	74	74	—	—	—	12	11	—	3.3	—	1.5	*P* < 0.05 H
M.S. Beeri, 2008	—	—	—	111	137	124	124	—	—	—	—	*P* < 0.05 H
Hannah Bruehl, 2009	59	60	15	42	46	41	47	—	—	—	—	---- H
Xiao-bing Zhou, 2012	73	73	—	252	280	162	370	81		66		*P* < 0.05 H
Qiong-yu Zhang, 2012	62	62	9	—	—	81	103	25.34	4.72	23.42	5.50	*P* < 0.05 H
Xiao-hong Zhao, 2009	65	65	—	—	—	31	71	17.46	9.51	10.19	6.09	*P* < 0.001 H
Jun-yi Wu, 2013	71	71	—	93	48	60	81	38		25		*P* < 0.05
Sheng Huang, 2015	65	65	—	49	51	58	42	41		18		*P* < 0.05
Jill K. Morris, 2014	76	74	16	165	99	97	167	—		—		*P* = 0.009 H

*Note*. Ex: experimental group; HC: healthy control group; HCs: healthy control subjects; Edu: average years of education of all participants; H: high insulin levels in patients in the experimental group; L: low insulin levels in patients in the experimental group.

## Abbreviations

A*β*42: Beta-amyloid 42
AD: Alzheimer's disease
APP: Amyloid precursor protein
CSF: Cerebrospinal fluid
ESs: Effect sizes
HC: Healthy control
HCs: Healthy control subjects
L: Light/mild
MH: Middle and heavy
MCI: Mild cognitive impairment
MMSE: Mini-Mental State Examination
NOS: Newcastle-Ottawa Scale
PRISMA: Reporting Items for Systematic Reviews and Meta-Analysis
s.d.: Standard deviation
VD: Vascular dementia
95% CI: 95% confidence interval.

## Additional Points

*Availability of Data and Material*. The datasets supporting the conclusions of this article are included within the article and its additional files.

## Supplementary Materials

Supplementary 1 Sensitivity analysis of differences in insulin levels in the CSF between nonpsychotic dementia patients and HC subjects.

Supplementary 2 Sensitivity analysis of the random-effects meta-analysis of differences in MMSE scores between patients with abnormal insulin levels and HC subjects.

Supplementary 3 Sensitivity analysis of differences in blood insulin levels between nonpsychotic dementia patients and HC subjects.

Supplementary 4 Sensitivity analysis of differences in blood insulin levels between vascular dementia (VD) patients and HC subjects.

Supplementary 5 Sensitivity analysis of differences in blood insulin levels between AD patients and HC subjects.

Supplementary 6 Sensitivity analysis of differences in insulin levels between L (light/mild) nonpsychotic dementia patients and HC subjects.

Supplementary 7 Sensitivity analysis of differences in insulin levels between MH (moderate to heavy) nonpsychotic dementia patients and HC subjects.

Supplementary 8 Publication bias in MMSE scores between patients with abnormal insulin levels and HC subjects.

Supplementary 9 Publication bias in insulin levels between nonpsychotic dementia patients and HC subjects.

Supplementary 10 Publication bias in insulin levels between AD patients and HC subjects.

Supplementary 11 PRISMA 2009 checklist.

Supplementary 12 PRISMA 2009 flow diagram.
