# Supplementary figures and images for: Relationship between Insulin Levels and Nonpsychotic Dementia: A Systematic Review and Meta-Analysis
Source: Neural Plast. 2017 Dec 27;2017:1230713. doi: 10.1155/2017/1230713 (PMC5763205; doi:10.1155/2017/1230713)

# Meta Analysis

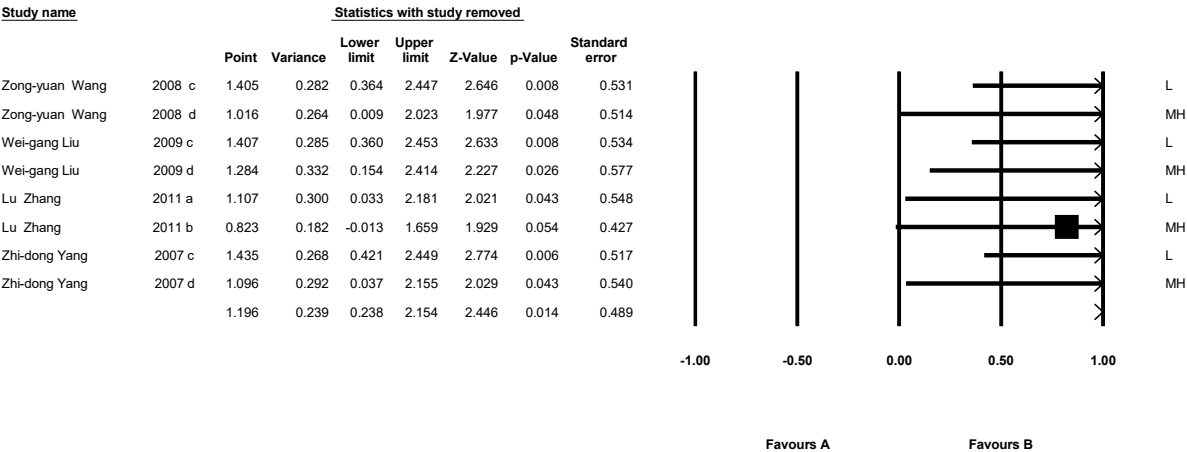

Supplement: Supplementary 1 — Sensitivity analysis of differences in insulin levels in the CSF between nonpsychotic dementia patients and HC subjects. [file 1230713.f1.pdf]

# Meta Analysis

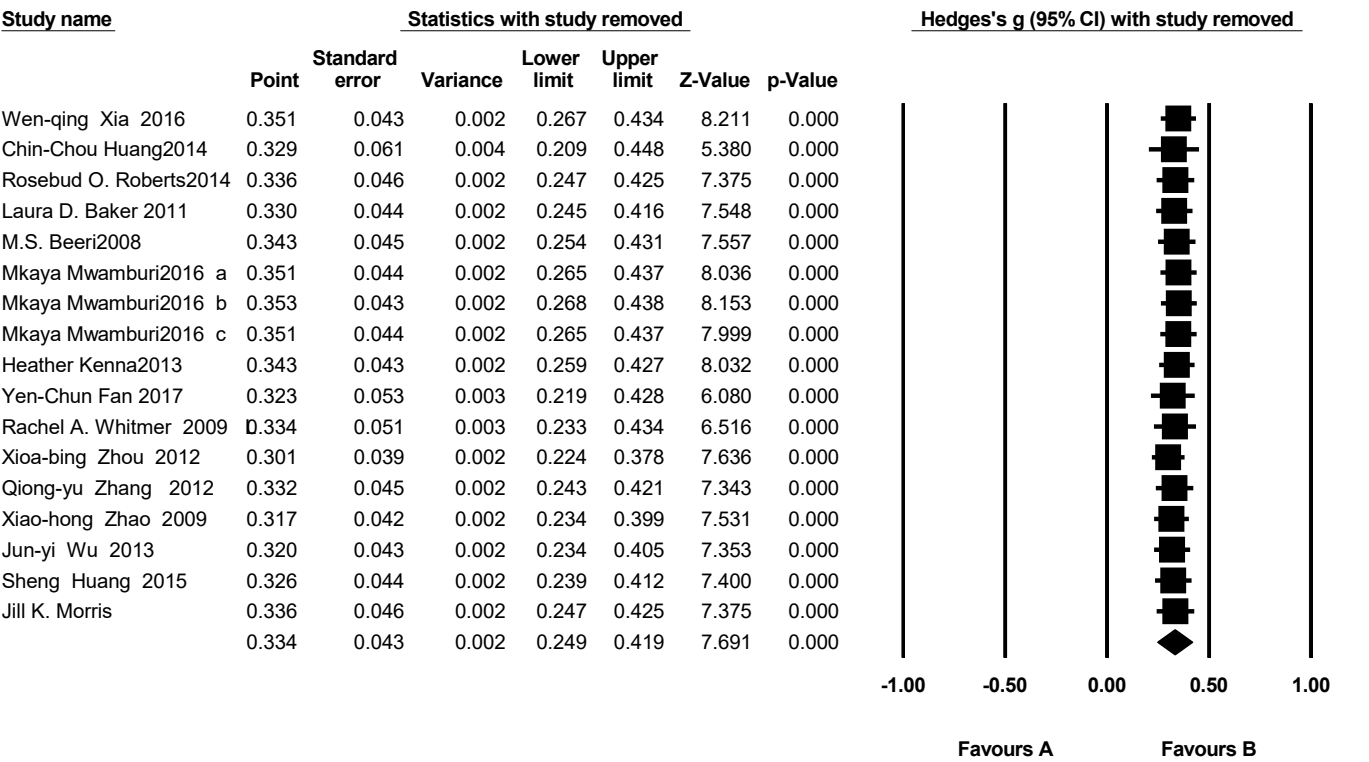

Supplement: Supplementary 2 — Sensitivity analysis of the random-effects meta-analysis of differences in MMSE scores between patients with abnormal insulin levels and HC subjects. [file 1230713.f2.pdf]

# Meta Analysis

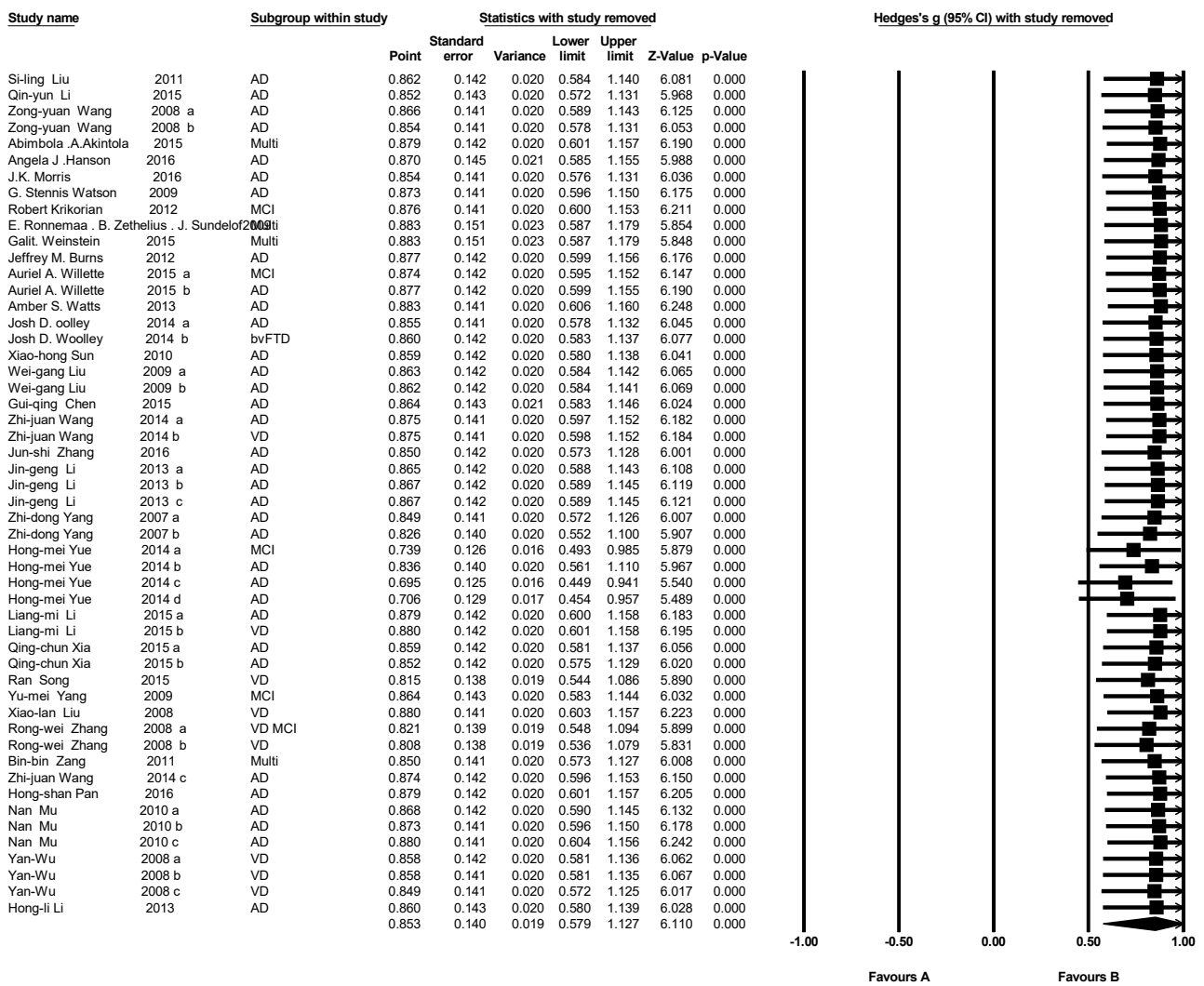

Supplement: Supplementary 3 — Sensitivity analysis of differences in blood insulin levels between nonpsychotic dementia patients and HC subjects. [file 1230713.f3.pdf]

# Meta Analysis

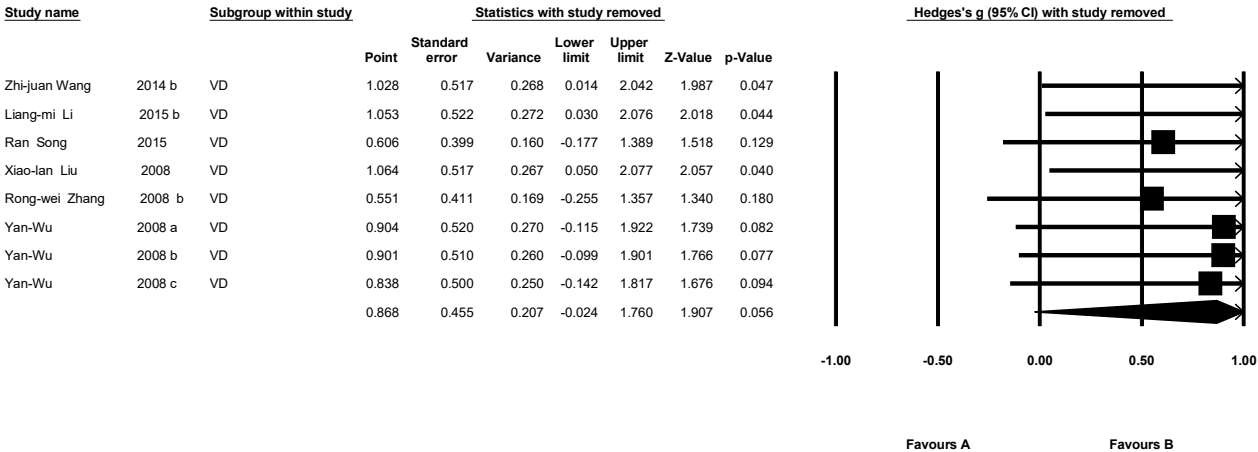

Supplement: Supplementary 4 — Sensitivity analysis of differences in blood insulin levels between vascular dementia (VD) patients and HC subjects. [file 1230713.f4.pdf]

# Meta Analysis

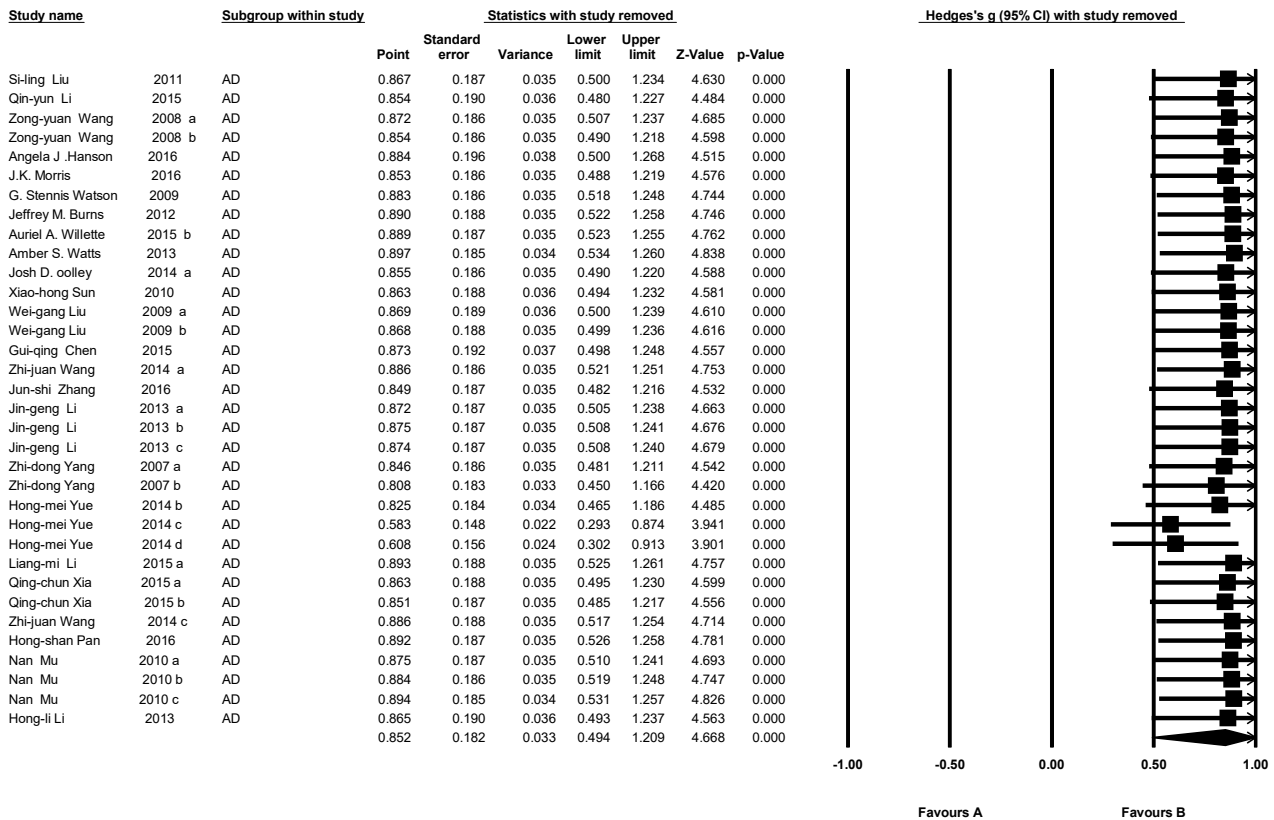

Supplement: Supplementary 5 — Sensitivity analysis of differences in blood insulin levels between AD patients and HC subjects. [file 1230713.f5.pdf]

# Meta Analysis

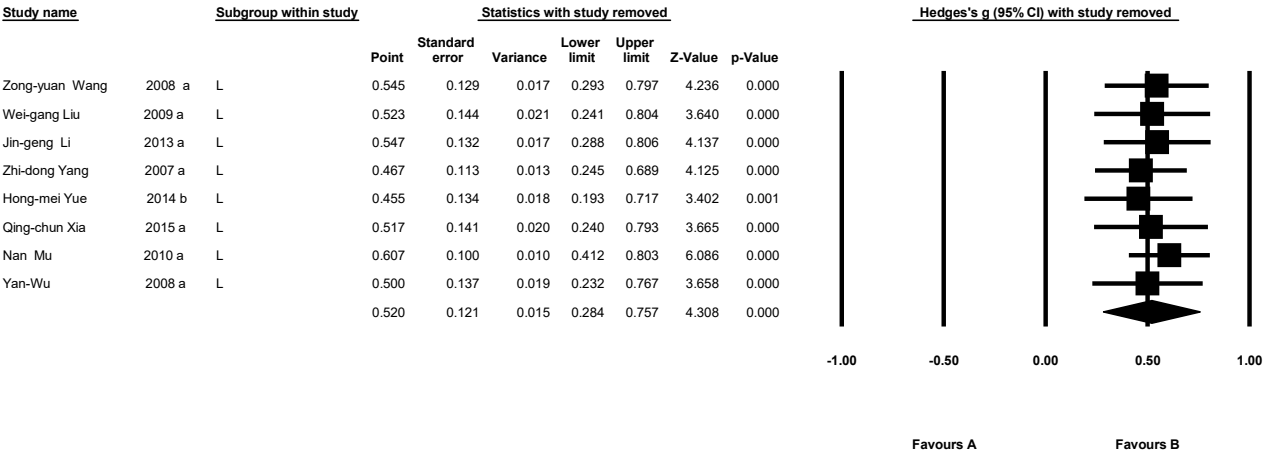

Supplement: Supplementary 6 — Sensitivity analysis of differences in insulin levels between L (light/mild) nonpsychotic dementia patients and HC subjects. [file 1230713.f6.pdf]

# Meta Analysis

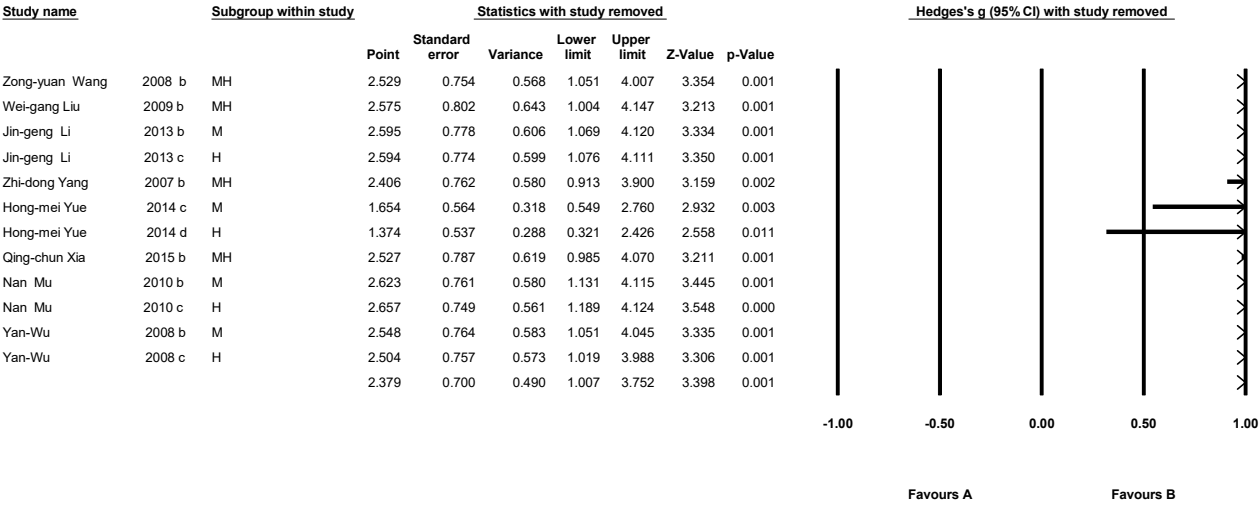

Supplement: Supplementary 7 — Sensitivity analysis of differences in insulin levels between MH (moderate to heavy) nonpsychotic dementia patients and HC subjects. [file 1230713.f7.pdf]

# Funnel Plot of Standard Error by Hedges's g

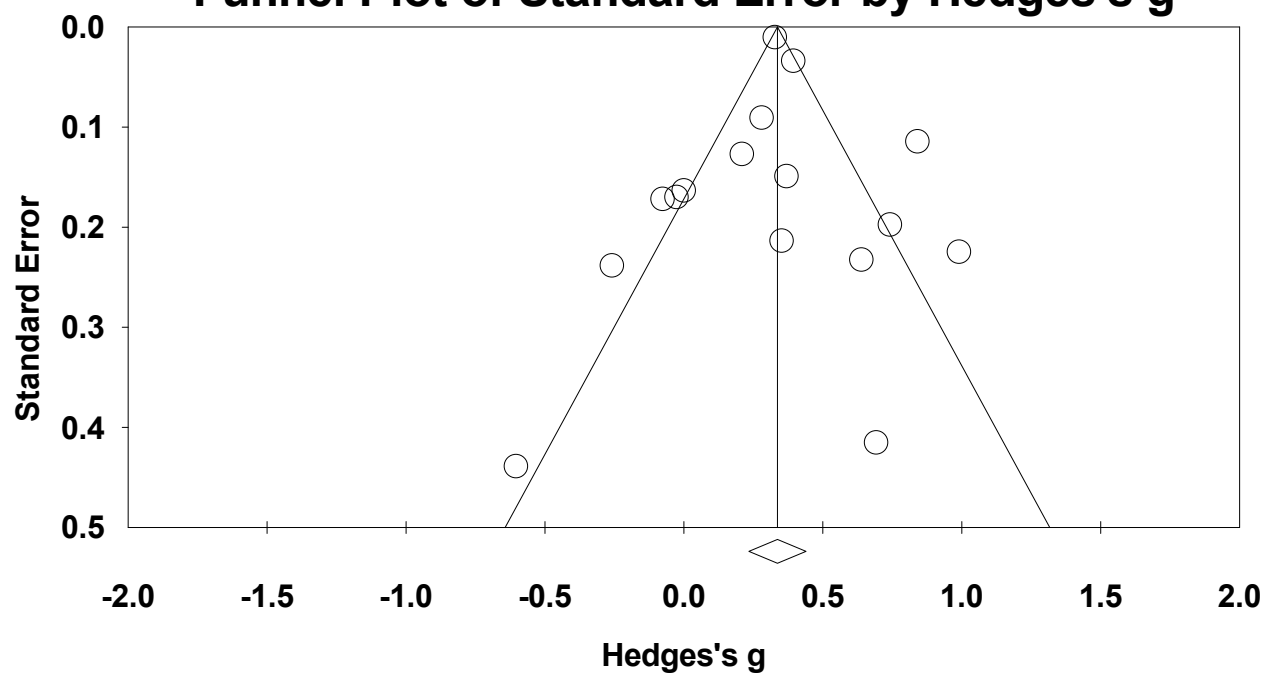

Supplement: Supplementary 8 — Publication bias in MMSE scores between patients with abnormal insulin levels and HC subjects. [file 1230713.f8.pdf]

**Funnel Plot of Standard Error by Hedges's g**

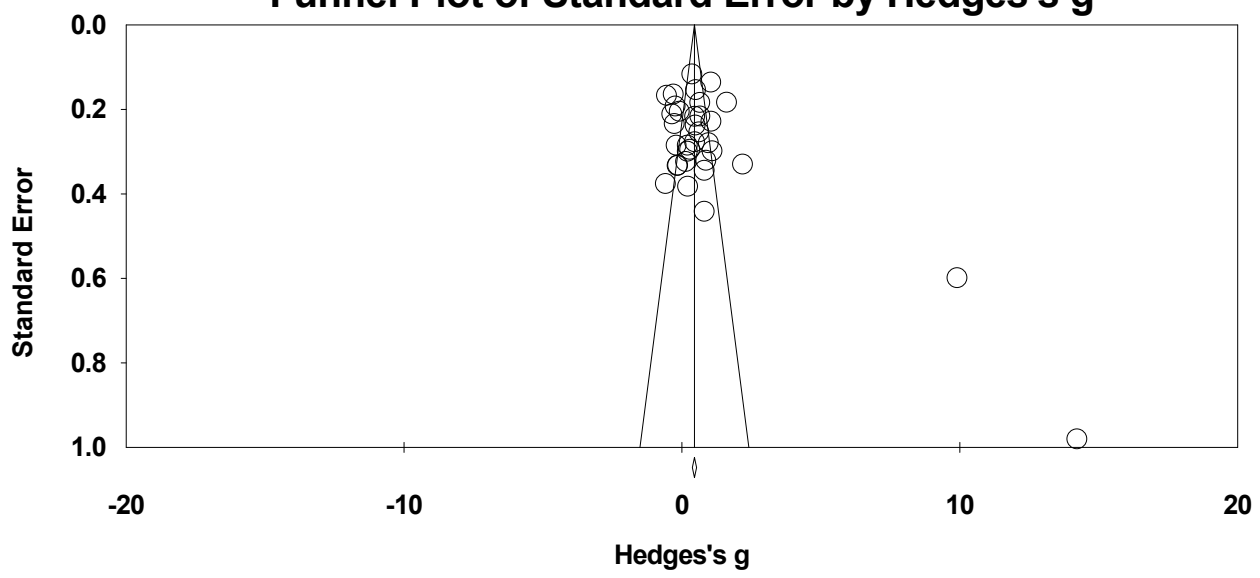

Supplement: Supplementary 9 — Publication bias in insulin levels between nonpsychotic dementia patients and HC subjects. [file 1230713.f9.pdf]

**Funnel Plot of Standard Error by Std diff in means**

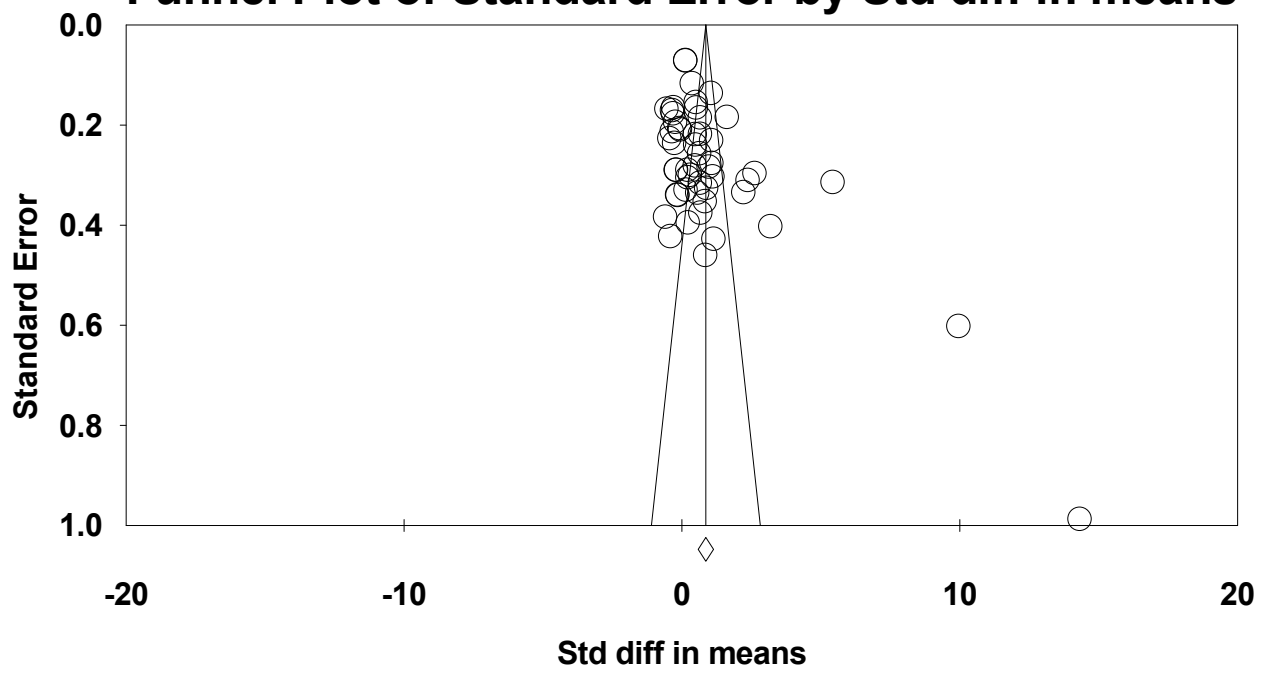

Supplement: Supplementary 10 — Publication bias in insulin levels between AD patients and HC subjects. [file 1230713.f10.pdf]
