# Supplementary material for: Relationship between Insulin Levels and Nonpsychotic Dementia: A Systematic Review and Meta-Analysis
Source: Neural Plast. 2017 Dec 27;2017:1230713. doi: 10.1155/2017/1230713 (PMC5763205; doi:10.1155/2017/1230713)
Supplement: Supplementary 11 — PRISMA 2009 checklist. [file 1230713.f11.doc]

| **Section/topic** | **#** | **Checklist item** | **Reported on page #** |
| --- | --- | --- | --- |
| **TITLE** | | |  |
| Title | 1 | Identify the report as a systematic review, meta-analysis, or both.  Both. | 5 |
| **ABSTRACT** | | |  |
| Structured summary | 2 | Provide a structured summary including, as applicable: background; objectives; data sources; study eligibility criteria, participants, and interventions; study appraisal and synthesis methods; results; limitations; conclusions and implications of key findings; systematic review registration number.  **Background：** Many scholars currently believe that insulin is involved in cognitive and memory functions. However, clinical observations have found inconsistent results in which insulin and non-psychotic dementia were positively correlated, negatively correlated and unrelated.  **Objectives：** To observe the relationship between non-psychotic dementia and insulin levels, we performed this meta-analysis.  **Data sources：** We included literature from several databases (PubMed, SCI, and Cochrane Library databases; CNKI, VIP, and Wanfang databases).  **Study eligibility criteria：** We enrolled the experimental or observational studies (case - control studies and cross - sectional studies) whose research objective were to explore the relationship between insulin levels and non-psychotic dementia; we excluded non-observational studies, unrelated research objective, psychotic dementia (depression, anxiety, schizophrenia, etc.), and drug intervention experiments.  **Participants：** All participants included non-psychiatric dementia patients, abnormal insulin levels patients, and the normal controls.  **Interventions：** Insulin levels are the intervention measures.  **Study appraisal and synthesis methods**： Comprehensive Meta-Analysis Version 2 software was used in this analysis. The sample size, mean, s.d. and events were primarily used to generate effect sizes (ESs) (the sample size and P-value were used in some studies in which the mean, s.d. and events were not available).  **Results：** The insulin levels in the cerebrospinal fluid from the non-psychotic dementia patients were decreased, while the levels in the peripheral blood were increased, and the Mini-Mental State Examination scores in the high insulin group were significantly lower than those in the healthy control (HC) group.  **Limitations：** Heterogeneity of this study was significant, although we had carried out the possibility, but more scientific experiments still need to be proved. In addition, the relationship between insulin and non-psychotic dementia in cerebrospinal fluid (CSF) and the relationship between insulin and MCI require a large number of related studies to verify, due to the small sample size.  **Conclusions and implications of key findings：** Blood insulin levels could be significantly increased in patients with non-psychotic dementia, and insulin levels could be used as an important indicator for clinical diagnosis of non-psychotic dementia.  **Systematic review registration number：**CRD42017069860. | 3-19 |
| **INTRODUCTION** | | |  |
| Rationale | 3 | Describe the rationale for the review in the context of what is already known.  Through the secondary analysis of enrolled studies with different sample sources and results, an objective and more credible basis about the relationship between insulin levels and non-psychotic dementia could be provided. | 3-5 |
| Objectives | 4 | Provide an explicit statement of questions being addressed with reference to participants, interventions, comparisons, outcomes, and study design (PICOS).  Experimental or observational studies of people with non-psychotic dementia or abnormal insulin levels who undertook no significant drug intervention with insulin levels or MMSE scores (events of non-psychotic dementia) as an outcome measures were enrolled in this study.  This study were divided into the following three parts according to the different study objectives: 1) the relationship between insulin levels in the CSF and the risk of non-psychotic dementia; 2) the variability in cognitive function scores (i.e., MMSE scores) by insulin levels in the peripheral blood; and 3) the differences in insulin levels between non-psychotic dementia and non-dementia patients (assuming that high insulin levels and non-psychotic dementia are positively correlated). | 5-6 |
| **METHODS** | | |  |
| Protocol and registration | 5 | Indicate if a review protocol exists, if and where it can be accessed (e.g., Web address), and, if available, provide registration information including registration number.  **The PROSPE：** Account: pqx1126@sina.com  Password: 19881126  **Systematic review registration number**：CRD42017069860 | 5-8 |
| Eligibility criteria | 6 | Specify study characteristics (e.g., PICOS, length of follow-up) and report characteristics (e.g., years considered, language, publication status) used as criteria for eligibility, giving rationale.  We selected all the published experimental or observational studies on the differences of insulin levels in patients with non-psychotic dementia and experimental or observational studies on differences of cognitive function in patients with abnormal insulin levels (Both of English and Chinese).  The length of follow-up was not limited.  According to the purpose of the system review, we need to incorporate as many studies as possible, so we collected all the relevant literatures in English and Chinese; but in order to ensure the reliability of the data source, we have only collected the published literature Data for unverified data. | 5-6 |
| Information sources | 7 | Describe all information sources (e.g., databases with dates of coverage, contact with study authors to identify additional studies) in the search and date last searched.  As long as there were significantly normal controls, all the follow-up studies meets the inclusion criteria will be included in this study regardless of the tracking time.  We collected all the data from January 1, 2007 to March 1, 2017. | 5 |
| Search | 8 | Present full electronic search strategy for at least one database, including any limits used, such that it could be repeated.  English data were searched from the PubMed, SCI, and Cochrane Library databases; Chinese data were searched from the CNKI, VIP, and Wanfang databases.  The searching items were Dementia # Alzheimer’s disease # AD # Mild cognitive impairment # MCI and Insulin # insulin signaling pathway in the abstract, key words or as the theme. All the relevant literatures were collected from January 1, 2007 to March 1, 2017. | 5 |
| Study selection | 9 | State the process for selecting studies (i.e., screening, eligibility, included in systematic review, and, if applicable, included in the meta-analysis).  We first excluded repeated literatures collected from the major databases; then, we excluded literatures (such as insulin like growth factor, expert experiences, etc.) that clearly did not meet the purpose of our study and obvious non-experimental and non-observational studies (review or research progress, conference papers, etc.), based on abstracts and titles of literatures; then, we read full text of the rest literatures for the second screening, and ruled out non-experimental and non-observational studies, literatures that were not associated with the insulin and dementia strictly; the included literatures were screened for the third time (excluded literatures that can’t be used to extract data because of un-consistent with our experimental purpose); finally, all the data of enrolled literatures was extracted.  At the end of each round of screening, the two reviewers and Yue-yun Liu converged literatures of the above round, and discussed the questionable or controversial literatures. | 5 |
| Data collection process | 10 | Describe method of data extraction from reports (e.g., piloted forms, independently, in duplicate) and any processes for obtaining and confirming data from investigators.  We printed the included literatures, the two reviewers grouped these articles from two aspects:   1. Non-psychotic dementia patients, healthy controls, insulin levels; 2. Abnormal insulin level patients, healthy controls, incidence of non-psychotic dementia/cognitive function (MMSE) scores.   The two reviewers marked the above data in the printed article, and then organized them into tables; Zhi-yi Yan helped to check the accuracy and integrity of these data and responsible for the data entry. | 7 |
| Data items | 11 | List and define all variables for which data were sought (e.g., PICOS, funding sources) and any assumptions and simplifications made.  The information including the first author’ name, sample size, gender and age of the subjects included, sample source, mean, s.d. and events as well as the p-values were included.  If the research data provided in the article was incomplete or only the final results were showed, literatures and their specific data were removed from this analysis.  In addition, all the studies that had common interest relationship with the supported funding resources were excluded. | 6 |
| Risk of bias in individual studies | 12 | Describe methods used for assessing risk of bias of individual studies (including specification of whether this was done at the study or outcome level), and how this information is to be used in any data synthesis.  Funnel plots were generated by plotting the ESs against the precision (inverse of the standard error) for each study and were used to visual inspect publication bias. The significance of the publication bias was determined using Egger’s test, which assesses the degree of asymmetry in funnel plots. Classic fail-safe N, which is an analysis of the number of missing (unpublished) studies that could increase the observed P-value to > 0.05, was also used to investigate publication bias. Statistical significance was set as a P-value < 0.05 unless indicated otherwise; P-values <0.1 were reported as trends.  The publication bias were demonstrated by a visual inspection of the funnel plots and confirmed by Egger’s tests in the non-psychotic dementia group, the insulin group and the AD group. | 6 |
| Summary measures | 13 | State the principal summary measures (e.g., risk ratio, difference in means).  The ESs were calculated as the standardized mean difference in insulin levels or MMSE scores between groups and converted to Hedge’s g, which provides an unbiased ES that is adjusted for the sample size. The 95% confidence interval (95% CI) was used to assess significant differences in the pooled ESs. | 7 |
| Synthesis of results | 14 | Describe the methods of handling data and combining results of studies, if done, including measures of consistency (e.g., I^2^) for each meta-analysis.  The Comprehensive Meta-Analysis Version 2 software (Biostat, Englewood, NJ, USA) was used for all statistical analyses. The sample size, mean, s.d. and events were primarily used to generate effect sizes (ESs) (the sample size and P-value were used in some studies in which the mean, s.d. and events were not available). The ESs were calculated as the standardized mean difference in insulin levels or MMSE scores between groups and converted to Hedge’s g, which provides an unbiased ES that is adjusted for the sample size. The 95% confidence interval (95% CI) was used to assess significant differences in the pooled ESs. Random-effects models were chosen for this meta-analysis because we hypothesized that within-study and between-study moderators would result in differences in the true ESs (94). We excluded one study at a time to determine whether the results were unduly secondary to a particular study. Data regarding the average age of the patients, the gender distribution of the patients, and the non-psychotic dementia classification and severity (i.e., MMSE) were also extracted.  Statistical differences in heterogeneity across studies were assessed using Cochran’s Q test (95), and statistical significance was set at P < 0.1, which prompted the presence of between-study heterogeneity. Inconsistencies across studies were determined by the I2 index, which evaluates the impact of the heterogeneity. An I2 of 0.25, 0.50 and 0.75 indicates small, moderate and high levels of heterogeneity, respectively. We then performed unrestricted maximum-likelihood random-effects meta-regressions of the ESs (96) to determine whether the covariates, including age, gender distribution (proportion of males) and the MMSE scores, served as moderators that influence the ESs. We then performed unrestricted maximum-likelihood random-effects meta-regressions of the ESs. | 7 |

Page 1 of 2

| **Section/topic** | **#** | **Checklist item** | **Reported on page #** |
| --- | --- | --- | --- |
| Risk of bias across studies | 15 | Specify any assessment of risk of bias that may affect the cumulative evidence (e.g., publication bias, selective reporting within studies).  As the ESs of the small sample studies estimate large variation, the appearance of extreme ES values is more likely to emerge than in large sample studies. If the publication bias was due to the small sample studies and was not statistically significant, Duval and Tweedie’s Trim and Fill method were applied for further analyses. | 8 |
| Additional analyses | 16 | Describe methods of additional analyses (e.g., sensitivity or subgroup analyses, meta-regression), if done, indicating which were pre-specified.  We excluded one study at a time to determine whether the results were unduly secondary to a particular study in each group. Sub-group analyses were performed considering class of the samples (VD or AD) and the degree of non-psychotic dementia in the patients. | 7-8 |
| **RESULTS** | | |  |
| Study selection | 17 | Give numbers of studies screened, assessed for eligibility, and included in the review, with reasons for exclusions at each stage, ideally with a flow diagram.  The initial search generated 1287 records with 474 English records and 813 Chinese records. After screening the titles and abstracts, 263 appropriate articles (including 120 English papers and 143 Chinese papers) related to the present subject were selected for full-text scrutiny. Original studies that reported data on peripheral blood and CSF insulin concentrations in at least two groups of subjects (Dementia or AD and MCI) were included. After further evaluating the 135 articles, 50 articles were included in the study, and the other 85 articles were excluded.  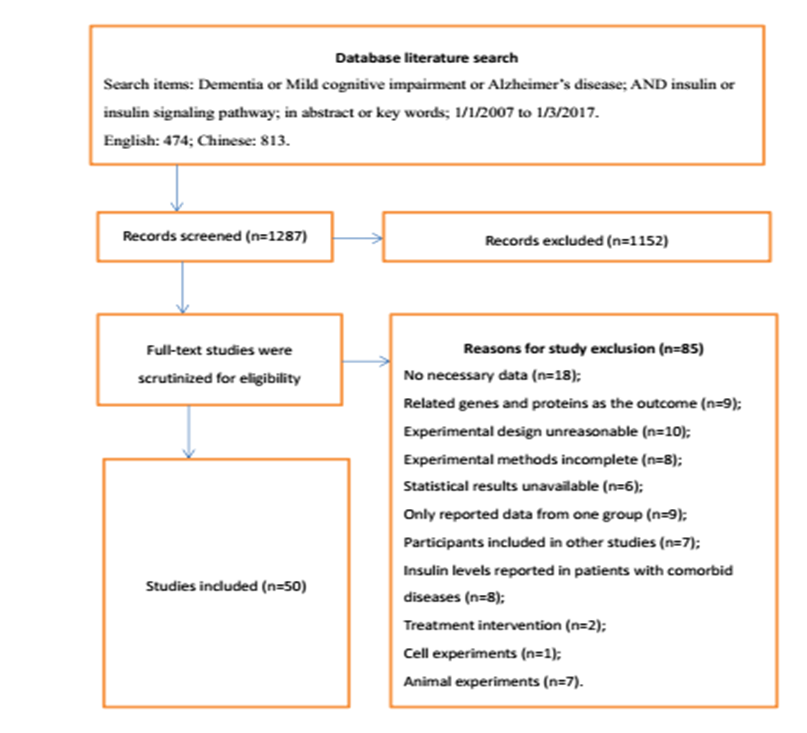 | 6 |
| Study characteristics | 18 | For each study, present characteristics for which data were extracted (e.g., study size, PICOS, follow-up period) and provide the citations.  Data regarding the average age of the patients, the gender distribution of the patients, sample size, mean and standard deviation (s.d.), events and P-values, the non-psychotic dementia classification and severity (i.e., MMSE) were also extracted. | 7 |
| Risk of bias within studies | 19 | Present data on risk of bias of each study and, if available, any outcome level assessment (see item 12).  In the abnormal insulin group, no significant publication bias was detected among the studies as demonstrated by a visual inspection of the funnel plots and confirmed by Egger’s tests; meanwhile, the non-psychotic dementia group and AD group showed slight publication biases with the following Egger’s test values.  The classic fail-safe N was used to assess the publication bias and revealed that 4805 missing studies for insulin and 1809 missing studies for AD would be required for the P-value to be > 0.05, supporting that the publication bias was unlikely to be the cause of the positive results of our meta-analysis. | 15 |

| Results of individual studies | 20 | For all outcomes considered (benefits or harms), present, for each study: (a) simple summary data for each intervention group (b) effect estimates and confidence intervals, ideally with a forest plot.  The non-psychotic dementia patients had significantly decreased insulin levels in the CSF compared with those in the HCs (Hedges’ g = 0.273, 95% CI = -2.189 to -0.340, P = 0.000).  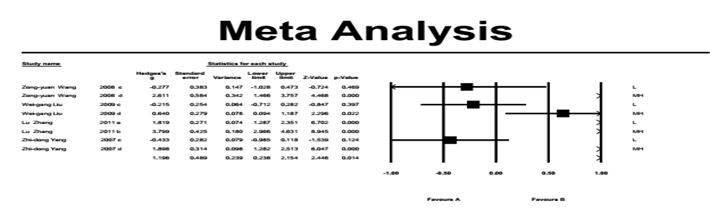  High insulin levels could increase the risk of non-psychotic dementia compared with the risk for HCs (Hedges’ g = 0.334, 95% CI = 0.249 to 0.419, P = 0.000).  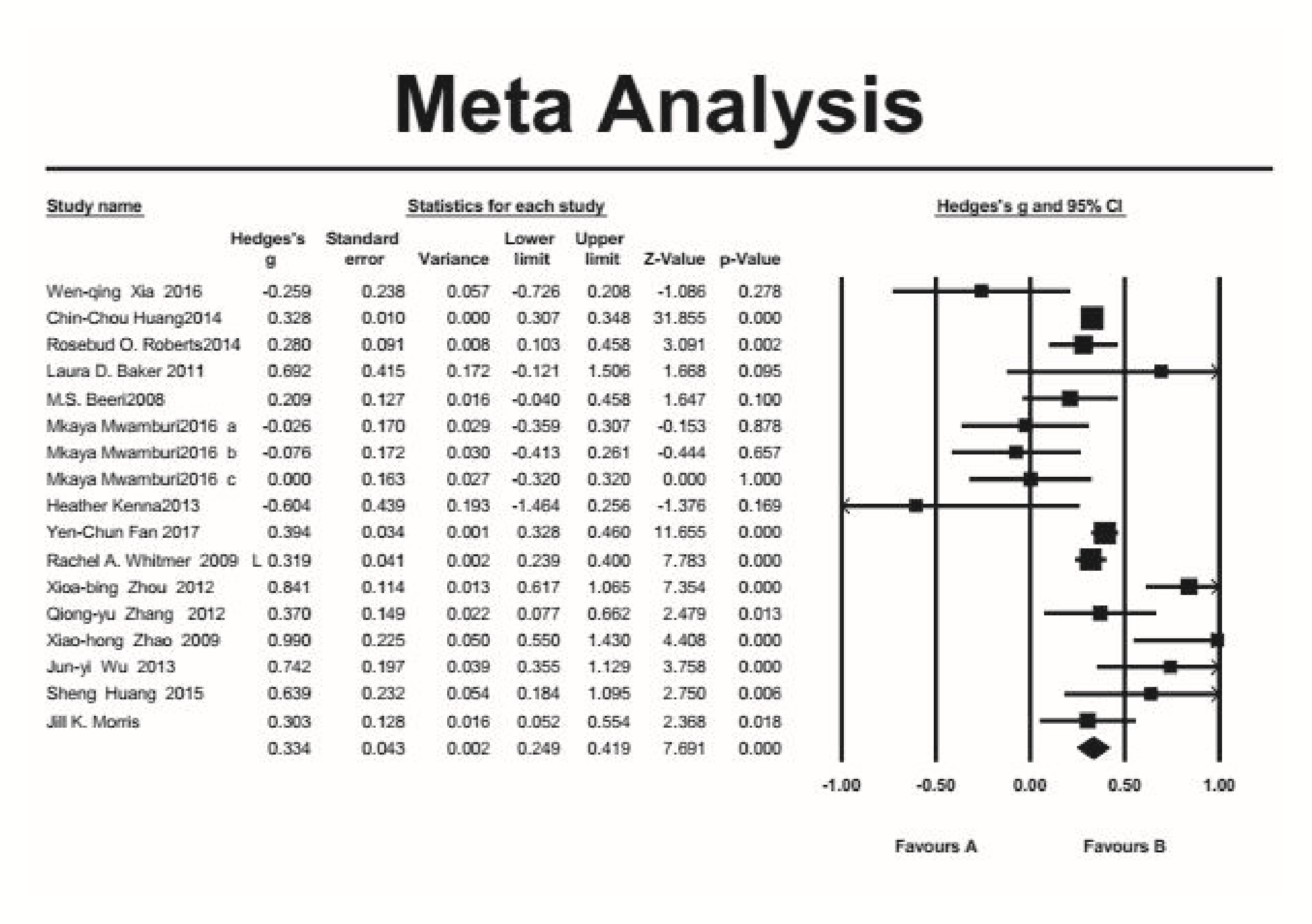  Non-psychotic dementia patients had significantly increased insulin levels compared with those of the HCs (Hedges' g = 0.853, 95% CI = 0.579 to 1.127, P = 0.000)  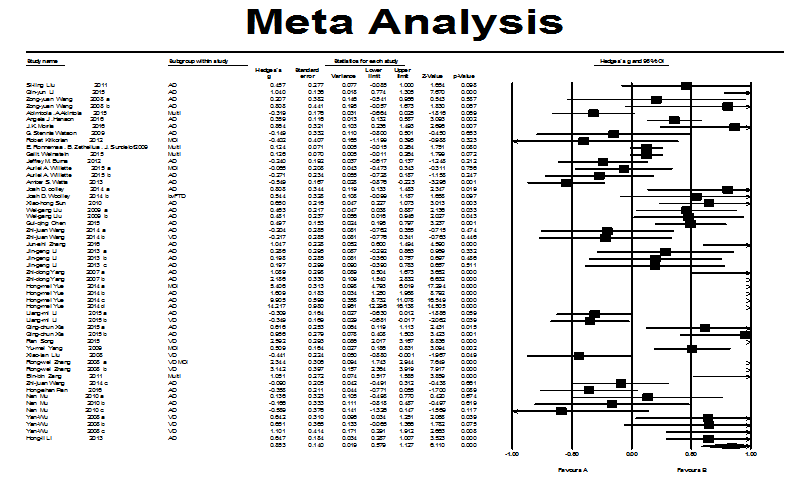 | 9-10 |
| --- | --- | --- | --- |
| Synthesis of results | 21 | Present results of each meta-analysis done, including confidence intervals and measures of consistency.  Our results showed that compared with the levels in the HC group, the CSF insulin levels decreased in patients with non-psychotic dementia, the insulin levels in the peripheral blood suggested an increasing trend, and high insulin levels in the peripheral blood were associated with reduced cognitive function scores. Significant heterogeneity was observed among the three studies in this meta-analysis (Q = 119.806, d.f. = 7, I2 = 94.157, P = 0.000); (Q = 64.967, d.f. = 15, I2 = 76.911, P = 0.000); (Q = 1184.942, d.f. = 51, I2 = 95.696, P = 0.000). | 9-10 |
| Risk of bias across studies | 22 | Present results of any assessment of risk of bias across studies (see Item 15).  In the non-psychotic dementia group, no significant publication bias was detected among the studies as demonstrated by a visual inspection of the funnel plots and confirmed by Egger’s tests (t = 0.054, d.f. = 14, P = 0.48); meanwhile, the insulin group and AD group showed slight publication biases with the following Egger’s test values: (for insulin: t = 3.26, d.f. = 50, P = 0.001; for AD: t = 2.399, d.f. = 32, P = 0.011). | 15 |
| Additional analysis | 23 | Give results of additional analyses, if done (e.g., sensitivity or subgroup analyses, meta-regression [see Item 16]).  Insulin levels were increased in the peripheral blood in the VD, AD, mild and middle to heavy non-psychotic dementia patients, and the CSF insulin levels were decreased in the non-psychotic dementia patients. The ES in the VD group was higher than that in the AD group, and the ES in the middle to heavy non-psychotic dementia group was higher than that in the mild non-psychotic dementia group. Meanwhile, our results showed no differences in the insulin levels between the MCI and non-psychotic dementia patients.  The sensitivity analysis indicated that our results were not unduly influenced by a particular study. | 9-10 |
| **DISCUSSION** | | |  |
| Summary of evidence | 24 | Summarize the main findings including the strength of evidence for each main outcome; consider their relevance to key groups (e.g., healthcare providers, users, and policy makers).  High insulin levels can be used as an important indicator for clinical diagnoses of non-psychotic dementia. | 18-19 |
| Limitations | 25 | Discuss limitations at study and outcome level (e.g., risk of bias), and at review-level (e.g., incomplete retrieval of identified research, reporting bias).  The enrolled sample number of insulin levels in CSF<10, the insulin levels in the MCI patients were not significantly different compared with those in the HCs, which requires more experimental data to verify that the blood insulin levels were increased in the MCI patients with a smaller difference than that in the non-psychotic dementia group. | 15-18 |
| Conclusions | 26 | Provide a general interpretation of the results in the context of other evidence, and implications for future research.  Several studies observed the insulin levels and MMSE scores in non-psychotic dementia patients without diabetes, type 2 DM patients without dementia, and non-psychotic dementia and type 2 diabetes patients and found no significant differences in age or sex. The insulin levels increased in the non-psychotic dementia patients, and the cognitive function scores decreased in the high insulin level subjects. Further investigations on insulin levels in MCI patients are necessary to confirm these results. | 15-18 |
| **FUNDING** | | |  |
| Funding | 27 | Describe sources of funding for the systematic review and other support (e.g., supply of data); role of funders for the systematic review.  This work was supported by the National Natural Science Foundation of China (grant numbers 81473597, 81630104), and 111 Project (No. B07007). |  |

*From:*  Moher D, Liberati A, Tetzlaff J, Altman DG, The PRISMA Group (2009). Preferred Reporting Items for Systematic Reviews and Meta-Analyses: The PRISMA Statement. PLoS Med 6(7): e1000097. doi:10.1371/journal.pmed1000097

For more information, visit: **www.prisma-statement.org**.

Page 2 of 2
