# Supplementary material for: Relationship between Insulin Levels and Nonpsychotic Dementia: A Systematic Review and Meta-Analysis
Source: Neural Plast. 2017 Dec 27;2017:1230713. doi: 10.1155/2017/1230713 (PMC5763205; doi:10.1155/2017/1230713)
Supplement: Supplementary 12 — PRISMA 2009 flow diagram. [file 1230713.f12.doc]

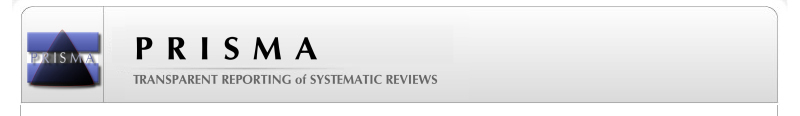
**PRISMA 2009 Flow Diagram**

**Screening**

**Included**

**Eligibility**

**Identification**

Records identified through database searching
(n =1287)

Additional records identified through other sources
(n =0)

Records after duplicates removed
(n =61)

Records screened
(n =1226)

Records excluded
(n =963)

Full-text articles assessed for eligibility
(n =263)

Full-text articles excluded, with reasons
(n =128)

Studies included in qualitative synthesis
(n =135)

Studies included in quantitative synthesis (meta-analysis)
(n =50)
